# A Doppler-exclusive non-invasive computational diagnostic framework for personalized transcatheter aortic valve replacement

**DOI:** 10.1038/s41598-023-33511-6

**Published:** 2023-05-17

**Authors:** Nikrouz Bahadormanesh, Benjamin Tomka, Mohamed Abdelkhalek, Seyedvahid Khodaei, Nima Maftoon, Zahra Keshavarz-Motamed

**Affiliations:** 1 Department of Mechanical Engineering, McMaster University, JHE-310, Hamilton, ON L8S 4L7 Canada; 2 School of Biomedical Engineering, McMaster University, Hamilton, ON Canada; 3 Department of Systems Design Engineering, University of Waterloo, Waterloo, ON Canada; 4 Centre for Bioengineering and Biotechnology, University of Waterloo, Waterloo, ON Canada; 5 School of Computational Science and Engineering, McMaster University, Hamilton, ON Canada

**Keywords:** Interventional cardiology, Cardiology, Engineering, Biomedical engineering

## Abstract

Given the associated risks with transcatheter aortic valve replacement (TAVR), it is crucial to determine how the implant will affect the valve dynamics and cardiac function, and if TAVR will improve or worsen the outcome of the patient. Effective treatment strategies, indeed, rely heavily on the complete understanding of the valve dynamics. We developed an innovative Doppler-exclusive non-invasive computational framework that can function as a diagnostic tool to assess valve dynamics in patients with aortic stenosis in both pre- and post-TAVR status. Clinical Doppler pressure was reduced by TAVR (52.2 ± 20.4 vs. 17.3 ± 13.8 [mmHg], p < 0.001), but it was not always accompanied by improvements in valve dynamics and left ventricle (LV) hemodynamics metrics. TAVR had no effect on LV workload in 4 patients, and LV workload post-TAVR significantly rose in 4 other patients. Despite the group level improvements in maximum LV pressure (166.4 ± 32.2 vs 131.4 ± 16.9 [mmHg], p < 0.05), only 5 of the 12 patients (41%) had a decrease in LV pressure. Moreover, TAVR did not always improve valve dynamics. TAVR did not necessarily result in a decrease (in 9 out of 12 patients investigated in this study) in major principal stress on the aortic valve leaflets which is one of the main contributors in valve *degeneration and, consequently, failure of heart valves*. Diastolic stresses increased significantly post-TAVR (34%, 109% and 81%, p < 0.001) for each left, right and non-coronary leaflets respectively. Moreover, we quantified the stiffness and material properties of aortic valve leaflets which correspond with the reduced calcified region average stiffness among leaflets (66%, 74% and 62%; p < 0.001; N = 12). Valve dynamics post-intervention should be quantified and monitored to ensure the improvement of patient conditions and prevent any further complications. Improper evaluation of biomechanical valve features pre-intervention as well as post-intervention may result in harmful effects post-TAVR in patients including paravalvular leaks, valve degeneration, failure of TAVR and heart failure.

## Introduction

Transcatheter aortic valve replacement (TAVR) is an emerging treatment alternative to surgical aortic valve replacement that covers a range of patients suffering from moderate to severe aortic stenosis (AS)^1^. AS is one of the most common and serious cardiovascular problems and if left untreated, often leads to death. Surgical valve replacement remains the standard treatment method for AS, however, many patients suffering from this pathology are at a high risk for surgery and may suffer death or other complications^2–4^. Up to 30% of patients with severe AS do not undergo surgical treatment due to the risks^5,6^, however, if left untreated AS carries dismal prognosis^7^. TAVR is a growing alternative for intervention of AS patients across a broad risk spectrum and has lower death rates in severe cases compared to a surgical approach^3,4^. Although TAVR has critical benefits for surgical high-risk patients, there are several drawbacks that patients may experience. Above 20% of patients suffer from paravalvular leaks post-intervention^1,8–10^, mitral regurgitation has been shown to occur in approximately 33%, and other negative outcomes such as heart failure have occurred^1,11^. Given the associated risks with TAVR, it is crucial to determine: how the implant will affect the cardiac function? When is the proper timing for intervention? Will TAVR improve or worsen the outcome of the patient^12^.

The condition of the heart valves heavily relies on the geometry and material properties of the leaflets as well as the interaction between the flow and the valve^13,14^. Valvular disease, including AS, often results in, or is a result of abnormal stress and strain distributions on aortic valve leaflets for both pre- and post-interventional cases^15,16^. The main cause of AS is calcification build-up^17^, however, additional potential causes include birth defects, rheumatic fever, or radiation therapy^18^. Mechanical strain and stress on the aortic valve are heavily influential on the progression of calcification^12,19–22^. The accurate estimate of the valve dynamics is crucial in the proper diagnosis of heart valve diseases^12,23–25^. Furthermore, the main causes of degeneration and, consequently, failure of prosthetic heart valves (e.g., transcatheter aortic valve) are mechanical stresses^26^. Effective treatment strategies, indeed, rely heavily on the complete understanding of the valve dynamics^27^. Such biomechanical features are greatly impactful when diagnosing and evaluating aortic valve pathologies^28^. However, there are no tools currently available to invasively or noninvasively quantify stress or strain distribution of aortic valve leaflets^24,29^.

Assessments of the valve dynamics in both pre- and post-TAVR can have incredible impacts on patient care. If available, assessment of valve dynamics provide valuable information about the patient’s state of cardiac deterioration as well as heart recovery, aiding in planning interventions and making critical clinical decisions with life-threatening risks^22,30^. Despite remarkable advancements in medical imaging, imaging on its own cannot provide valve dynamics features which are very important for the long-term health of the heart and durability of the valve leaflets^24,31^. There are no current clinical tools available to invasively or noninvasively quantify valve dynamics^24,29,31^.

Numerical frameworks devised for 3-D quantification of valve dynamics in patients are expected to have the following 6 requirements:

- Requirement #1. The physical model should represent the realistic dynamic behavior of aortic valve leaflets. The numerical solver utilized must be able to simulate the large deformations experienced by the anisotropic tissue of aortic valves during cardiac cycle imposed by transvalvular blood pressure. Failure to accurately address the leaflets anisotropic behaviors could result in an unreliable stress distribution and inaccurate leaflet displacement, in particular during the closure of the valves^32,33^.
- Requirement #2. Currently, there is an agreement amongst many researchers that valvular disease is a complex condition that also depends on the dictates of the ventricle and the vascular system^1,34–39^. Local flow dynamics are significantly impacted by both downstream and upstream conditions. It is therefore essential to impose correct boundary conditions to the model that takes the interactive coupling of the valve, ventricle, and the vascular system into account^1,40–43^.
- Requirement #3. Patient-specific material properties are integral to the accuracy of the model to mimic each patient’s pathology and tissue behavior. Though several experimental test have been performed on native leaflets, calcification on unhealthy leaflets change the behavior of the tissue and requires an additional calibration process to adjust the material properties^44–46^. Furthermore, because calcification patterns on aortic leaflets differ, each leaflet's dynamic behavior should be evaluated on a leaflet-by-leaflet basis^47–50^. This is especially crucial for patients with valvular disease who undergo transcatheter aortic valve replacement^51–53^.
- Requirement #4. The 3-D geometry of aortic valve leaflets should be patient-specific and reconstructed in both pre- and post-interventional states. Using simplified geometry, such as symmetric leaflets, would make the framework blind to geometrical differences of leaflets^54^. Leaflet sizes differ, and consequently experience different stress distribution and biomechanical behavior^55^. Additionally, patients have other geometrical parameters, including the height of aortic valve cusps, which can have a considerable impact on the stress distribution^54,55^. As there is a link between leaflets’ stress distributions and disease progressions^22,50^, it is critical to consider the patient-specific leaflet geometry when assessing the stress and biomechanical features.
- Requirement #5. A computational framework should ideally be transferrable to clinical practice. As such, it is imperative that the computational cost of the framework, non-invasiveness, and number of unknown parameters in each step of the framework are rigorously evaluated^42,56–58^. Considering the predetermined clinical goals, a balance must be considered between accuracy, time and invasiveness to obtain a feasible framework for clinical applications^59,60^.
- Requirement #6. Any computational framework devised for clinical diagnosis should be validated against clinical data, e.g., clinical cardiac catheterization, DE, MRI and/or CT^56,61–65^.

Many past studies have used nonlinear finite element methods to quantify stress and strain distributions on aortic valve leaflets. None of these previous models can satisfy all six of the above requirements^2,13,24,66–87^. Several past studies have been unable to satisfy Requirement 1 as quasi-static assumptions were used for motion equations, which assume a static situation rather than dynamic^75,83,85^ or isotropic hyper-elastic models were used which do not account for the anisotropic structure of the aortic valve leaflets^13,27,68,70,71,73,74,77,80,82,84^. Requirement 2 has never been met as all previous models have used constant or non-patient specific pressure loads which ultimately leads to the neglecting of Requirement 3 as material properties cannot be calibrated properly for each patient^2,13,27,66,67,69–73,76,78–80,82–85,88^. Several other studies have used simplified 3-D geometries for aortic valve leaflets that are symmetric which contradicts Requirement 4^27,67,78–81,84,86–88^. Requirements 5 and 6 have never been discussed comprehensively in any of the proposed models^2,13,27,66–88^.

In this study, we used computational mechanics as a powerful means to enhance clinical measurements, and medical imaging to develop a novel diagnostic method that can be eventually used for monitoring, treatment planning and risk assessment in patients with aortic stenosis in both pre- and post-TAVR states *upon future further validations*. These developments rely heavily on the data gathered from the various forms of medical imaging such as Doppler echocardiography (DE), computed tomography (CT), and magnetic resonance imaging (MRI). CT carries risk including exposure to ionizing radiation and MRI can not be used in patients with implanted devices, remaining a major risk during the examination. DE is a risk-free, non-invasive imaging technique commonly used in patients with cardiovascular diseases. In this study, we developed a highly innovative non-invasive Doppler-exclusive computational-mechanics framework that can function as a diagnostic tool to assess aortic valve dynamics in pre- and post-TAVR states at no risk to the patients. The developed diagnostic tool is able to dynamically couple the local valve dynamics with the global circulatory system which provides a platform for testing intervention scenarios (e.g. TAVR) and evaluating their effects. In order to achieve this, we developed a framework based on an innovative Doppler-based patient-specific lumped-parameter model and 3-D non-linear finite element solver that satisfies all of the 6 mentioned requirements for developing a clinically-effective computational diagnostic tool to quantify valve dynamics (e.g. transient 3-D distribution of stress and displacement, 3-D deformed shape of leaflets, geometric orifice area and angular positions of leaflets) in patients in both pre- and post-TAVR states. Our lumped-parameter model allows for the analysis of any combination of complex valvular, vascular and ventricular diseases in patients, purposefully uses limited and reliable non-invasive input parameters using Doppler echocardiography and sphygmomanometer to continuously calculate patient-specific local and global hemodynamics quantities^38^. To construct the 3-D geometry of the asymmetric aortic valve leaflets, a Doppler-based parametric method was developed. A multi-thread algorithm was used for solving linear system of equations of the finite element solver in a timely manner due to the importance of computational time in clinical applications. We used clinical data of 12 patients with AS in both pre- and post-TAVR states (24 cases) not only to validate the proposed framework but also to demonstrate its monitoring capacities by providing novel analyses and interpretations of clinical data. The validation was done against clinical Doppler echocardiography data and measurements.

## Methodology

**Figure 1:**
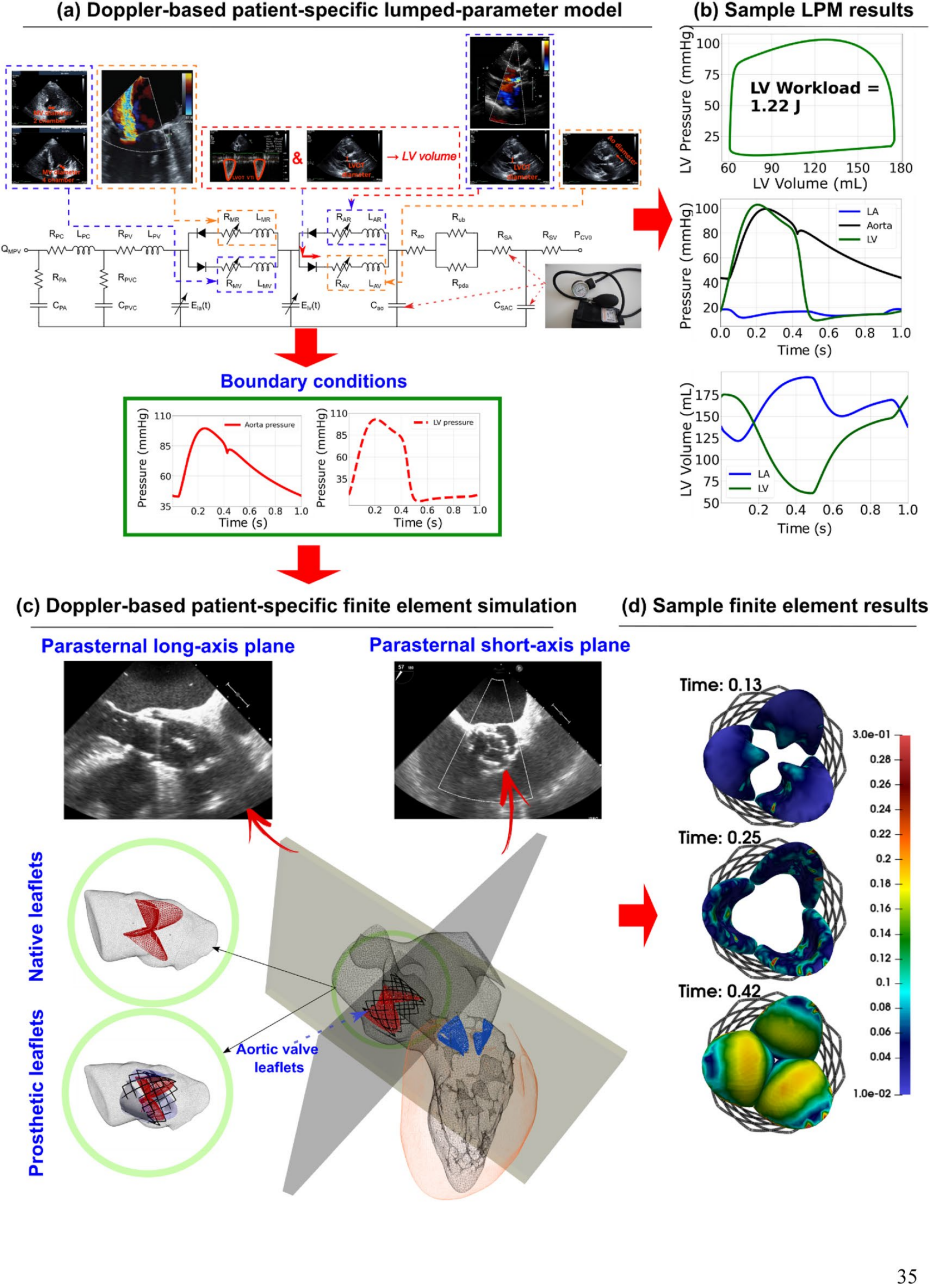
Schematic diagram of Doppler-based diagnostic framework (Doppler-based lumped-parameter model and Doppler-based 3-D non-linear finite element solver). (**a**) Schematic diagram of the lumped-parameter model which includes the following sub-models: (i) left ventricle; (ii) left atrium; (iii) aortic valve; (iv) mitral valve; (v) pulmonary circulation; and (vi) systemic circulation^38^. The lumped-parameter model provided us with patient-specific transient loads on the aortic valve, including the aorta and left ventricle pressure during cardiac cycle. These transient loads are imposed on both the ventricular and aortic surface of aortic valve leaflets; (**b**) sample curves representing global hemodynamic parameters including pressure–volume loop, volume, and pressure variation of different regions of the heart during full-cardiac cycle including the left ventricle, aorta, and left atrium. The workload is the integral of LV pressure and its volume change and was computed as the area encompassed by the LV pressure and volume loop; (**c**) Doppler heart views used for the valve reconstruction to be used in finite element simulations; (**d**) sample finite element results, including 3D distribution of stress and displacement over heart valve leaflets at different time points of the cardiac cycle.

**Figure 2:**
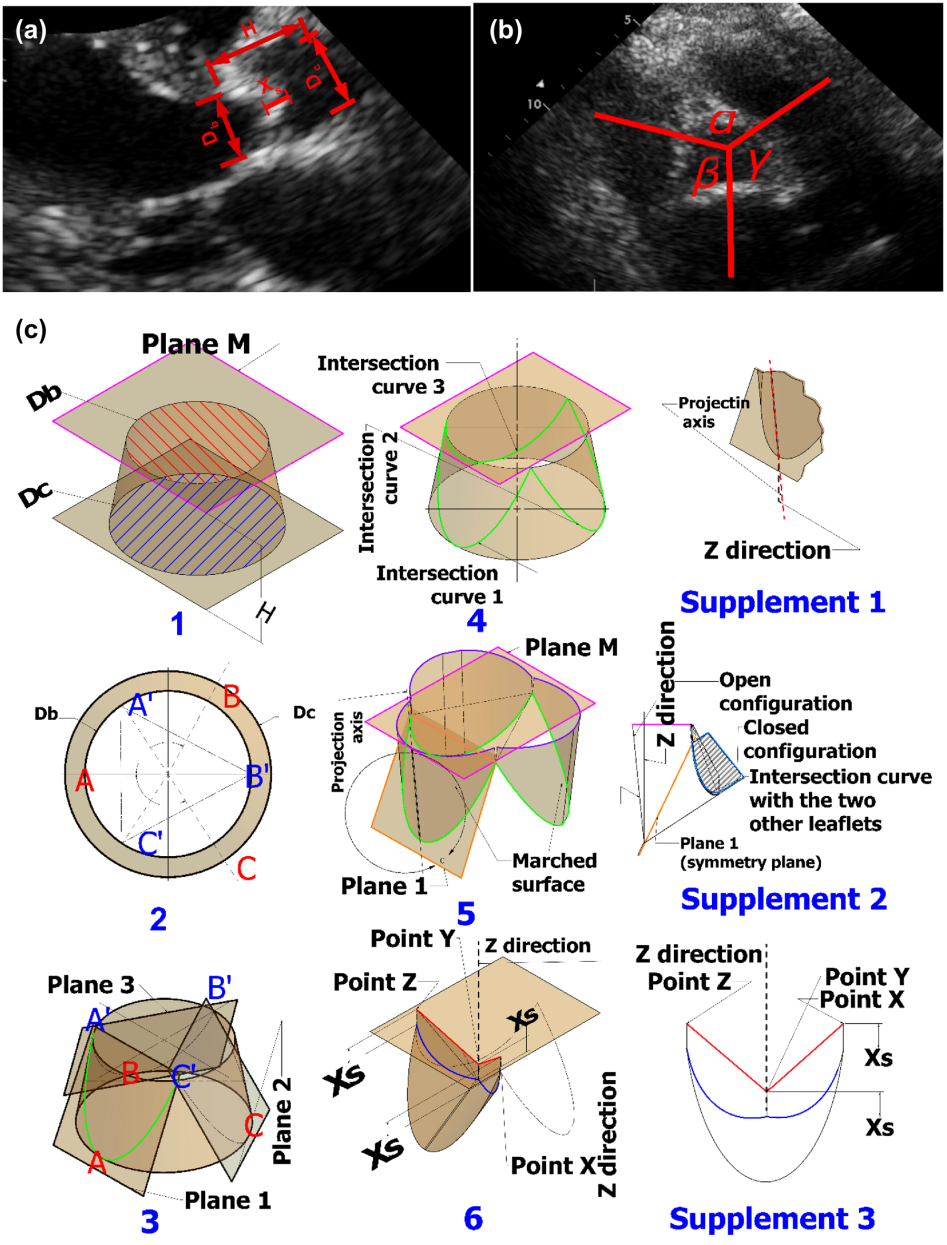
Doppler-based patient-specific 3-D geometry reconstruction of the aortic valve. (**a**) Parasternal long-axis Doppler echocardiographic image with labelled dimensions including base diameter (${D}_{b}$), diameter of commissures (${D}_{c}$), valve height ($H$), length of central coaptation (${X}_{s}$) to be used for valve reconstruction; (**b**) parasternal short-axis Doppler echocardiographic image with labelled dimensions (two angles of the leaflets; α and β); (**c**) patient-specific 3-D geometry construction of aortic valve leaflets. All input parameters are measured using parasternal long-axis view and parasternal short-axis view.

**Figure 3:**
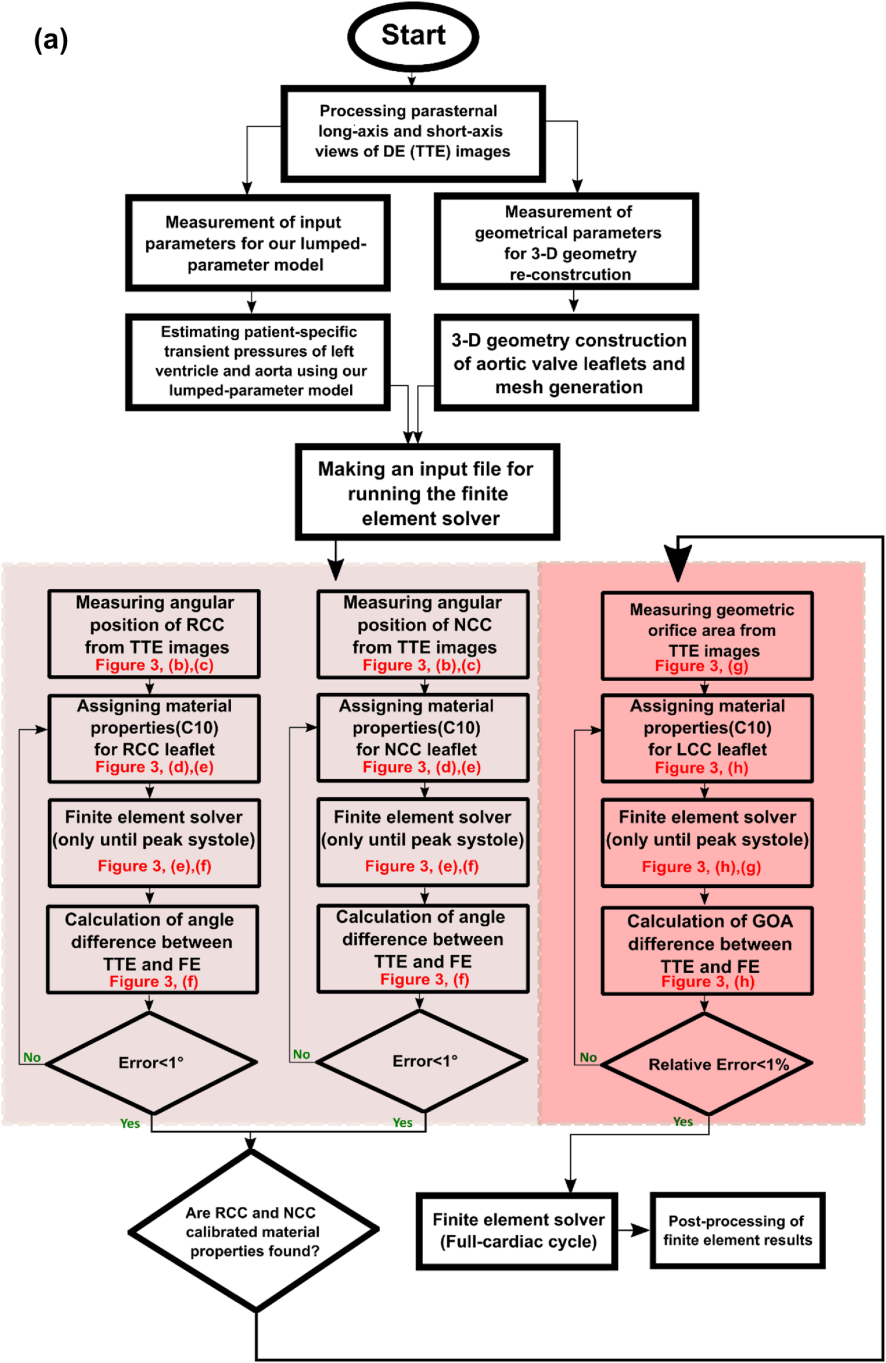

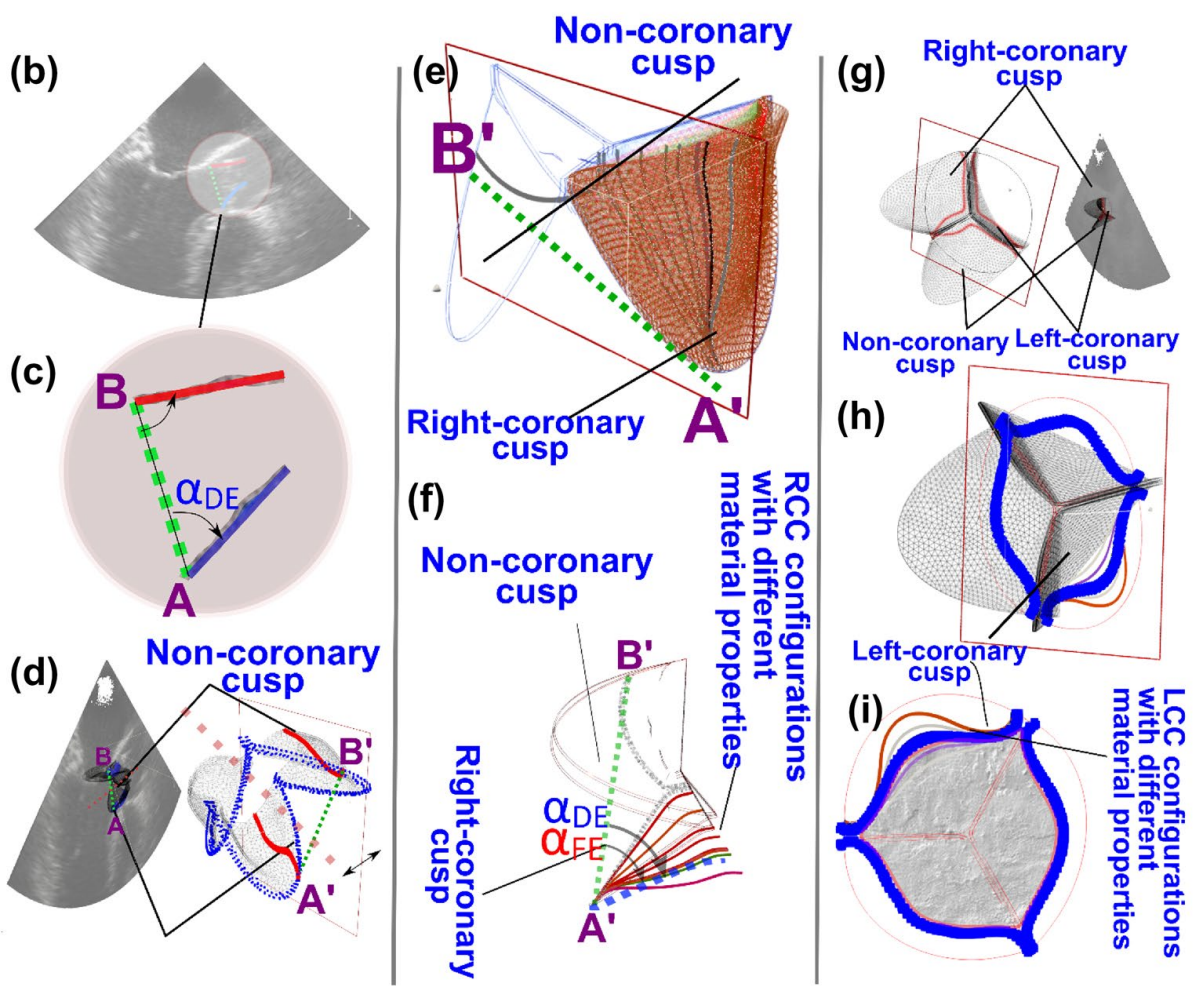
Doppler-based patient-specific lumped-parameter model and finite element solver flow chart. (**A**) As a fully non-invasive framework, all required parameters for LPM and 3D image construction are collected non-invasively using Doppler echocardiography images. Having transient pressure loads and 3-D geometry of aortic valve leaflets, a patient-specific finite element simulation was performed. To have a calibrated material properties for each leaflet, angular position of leaflets and geometric orifice area were considered. (**B**,**C**) Parasternal long-axis view Doppler echocardiography (TTE) images are used to manually measure the angular rotation of leaflets using the AB line (drawn between the attachment of the left coronary cusp and non-coronary cups to the aortic root). (**D**) The parasternal long-axis view is replicated in the computational domain. (**E**,**F**) Angular positions of leaflets (RCC and NCC) in the computational domain are matched with the measured angular positions in Doppler echocardiography images. (**G**) The geometric orifice area of the aortic valve is measured in the fully open configuration using parasternal short-axis view. (**H**,**I**) The stiffness of the left coronary cusp is matched to the geometric orifice area of the aortic valve.

**Figure 4:**
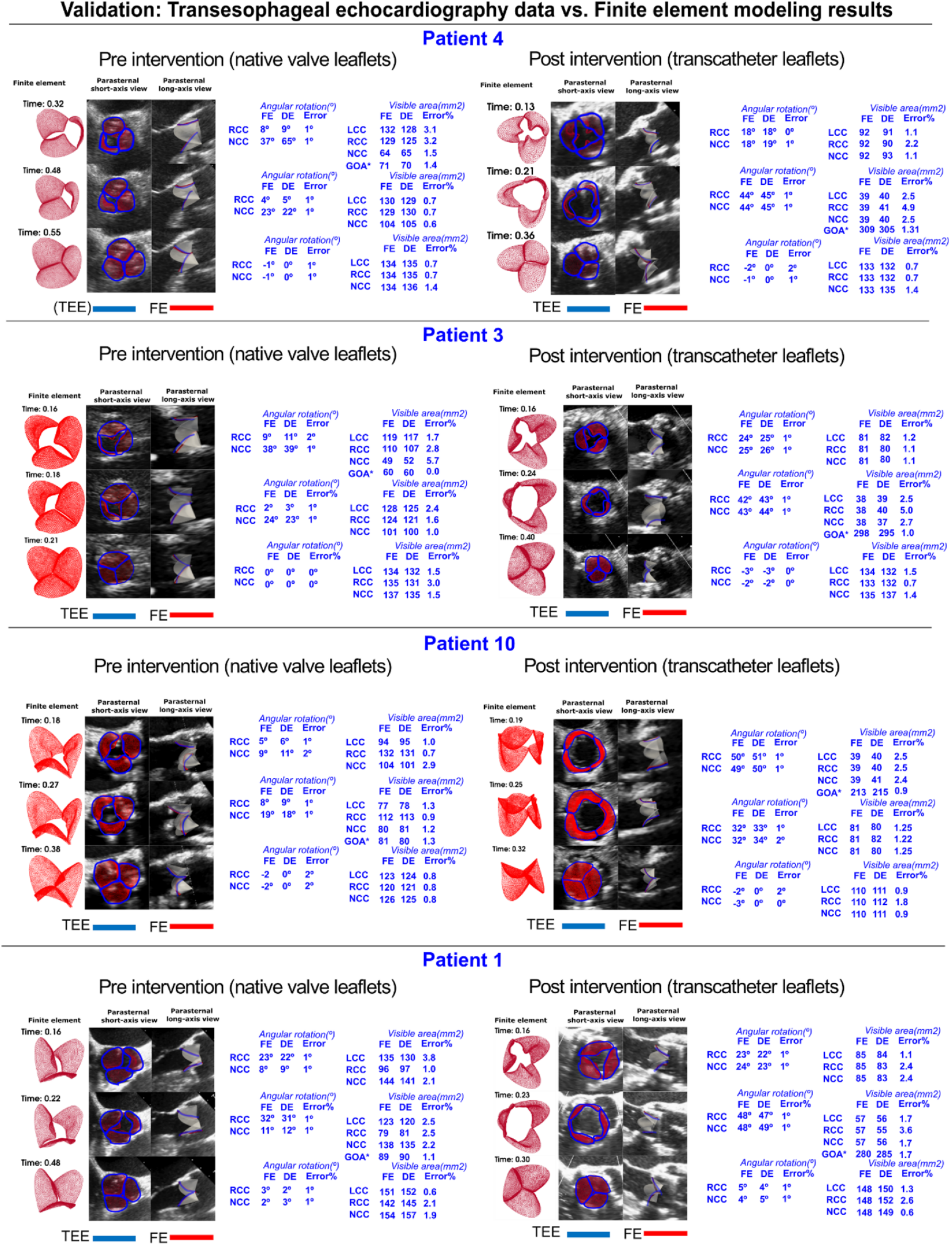
Validation: Doppler-based 3-D non-linear finite element solver vs*.* clinical transesophageal echocardiography data. The results of the finite element solver and high-quality TEE images have been compared geometrically in different time steps. The figures are shown in three time-steps throughout the cardiac cycle. Angular position was calculated using long axis-parasternal plane view and different visible surface area’s were determined in parasternal short-axis plane views. The mentioned quantitative values achieved through TEE are compared to the results of our Doppler-based framework both pre- and post-TAVR for patients #4, #3, #10, and #1.

**Figure 5:**
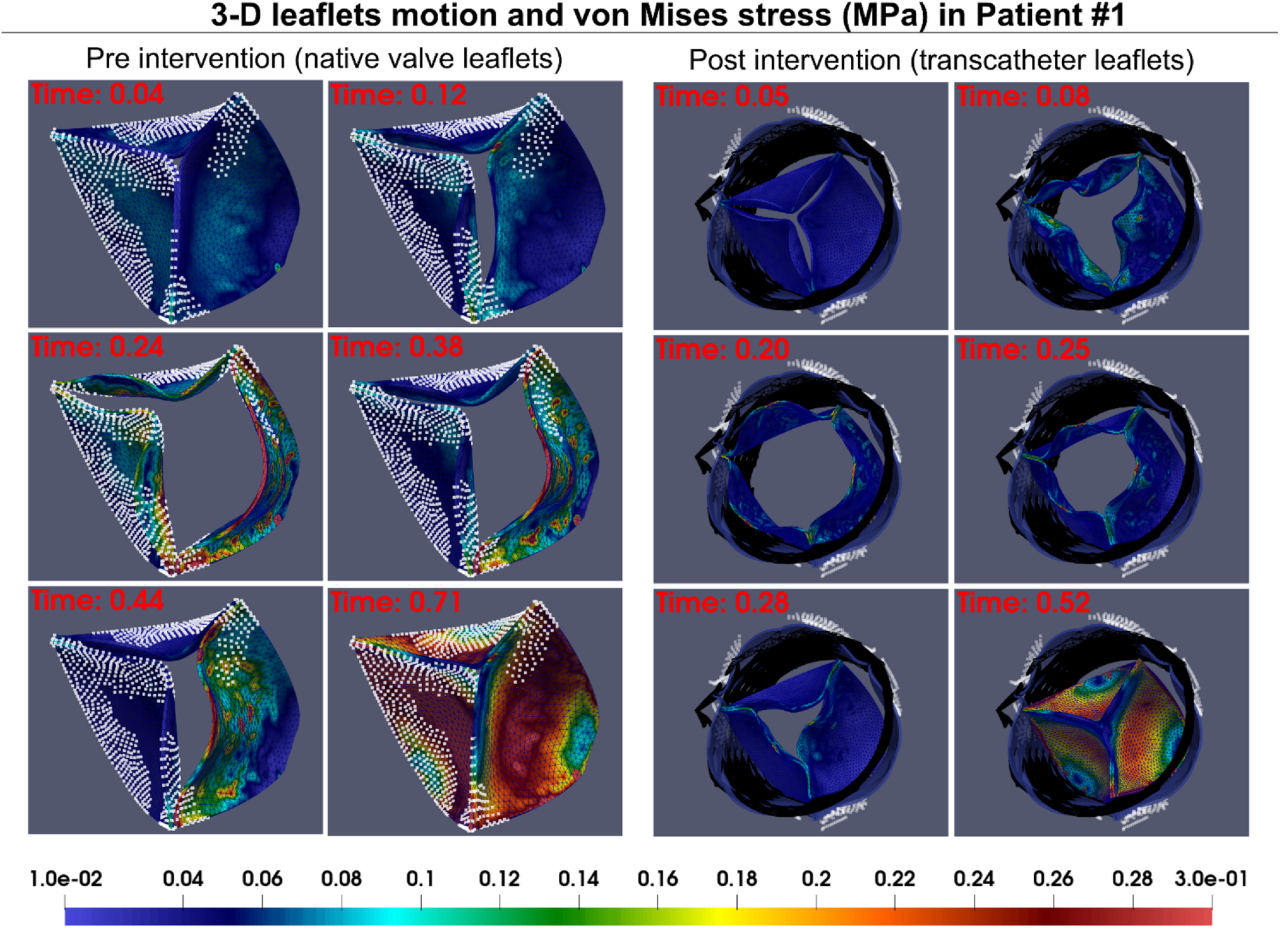
3D motion and 3D distribution contours of von Mises stress in patient #1 at six time points throughout the cardiac cycle in both pre- and post-intervention states. Using our framework, we estimated 3D deformation of aortic valve leaflet during full cardiac cycle as well as the von Mises stress distribution. The regions covered with white points are representing the calcified areas visualized manually by using multi-slice CT images.

**Figure 6:**
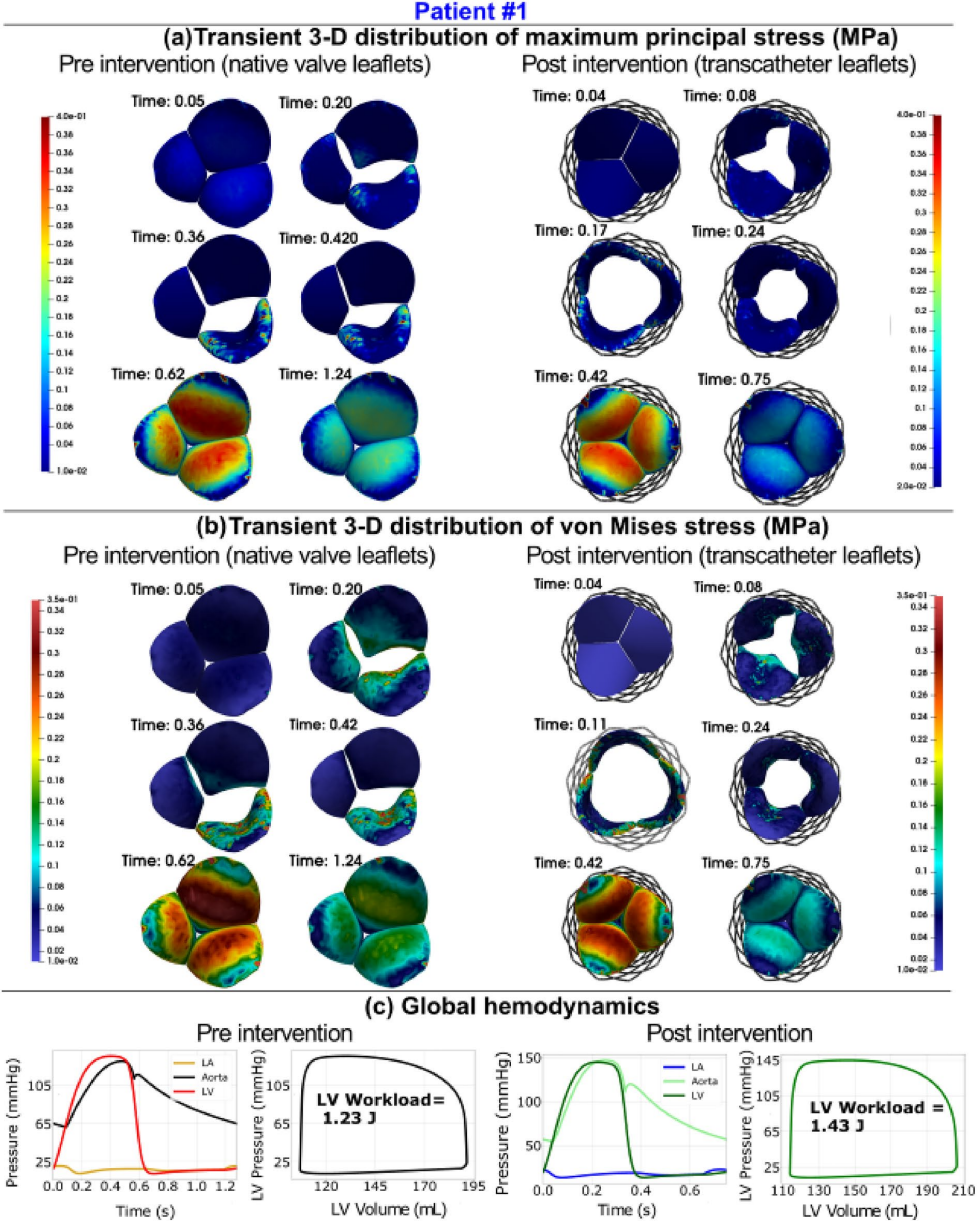
Changes in valve dynamics and global hemodynamics in patient #1 between baseline and 90-day post-TAVR. (**a**) Transient distribution of major principal stress at six time phases of the cardiac cycle: early systole (start of valve opening), early to peak systole (valve opening), peak systole (fully open), peak to late systole (valve closure), early diastole (closed configuration) and late diastole (closed configuration); (**b**) transient distribution of the von Mises stress at six time phases of the cardiac cycle: early systole(start of valve opening), early to peak systole(valve opening), peak systole (fully open), peak to late systole (valve closure), early diastole (closed configuration) and late diastole (closed configuration); (**c**) *Global hemodynamics*: LV workload; aorta and LV pressures in both pre- and post-intervention states. *Patient #1*: *Pre-TAVR*: severe aortic stenosis (EOA = 0.9 cm^2^), type 2 diabetes mellitus, coronary artery disease and hypertension, chronic AF, ejection fraction: 36%, brachial pressures: 58 and 132 mmHg; *Post-TAVR*: aortic valve (EOA = 2.0 cm^2^), hypertension, type 2 diabetes mellitus, coronary artery disease, paravalvular leakage, chronic AF, mild-moderate mitral regurgitation, ejection fraction: 56%, brachial pressures: 55 and 148 mmHg.

**Figure 7:**
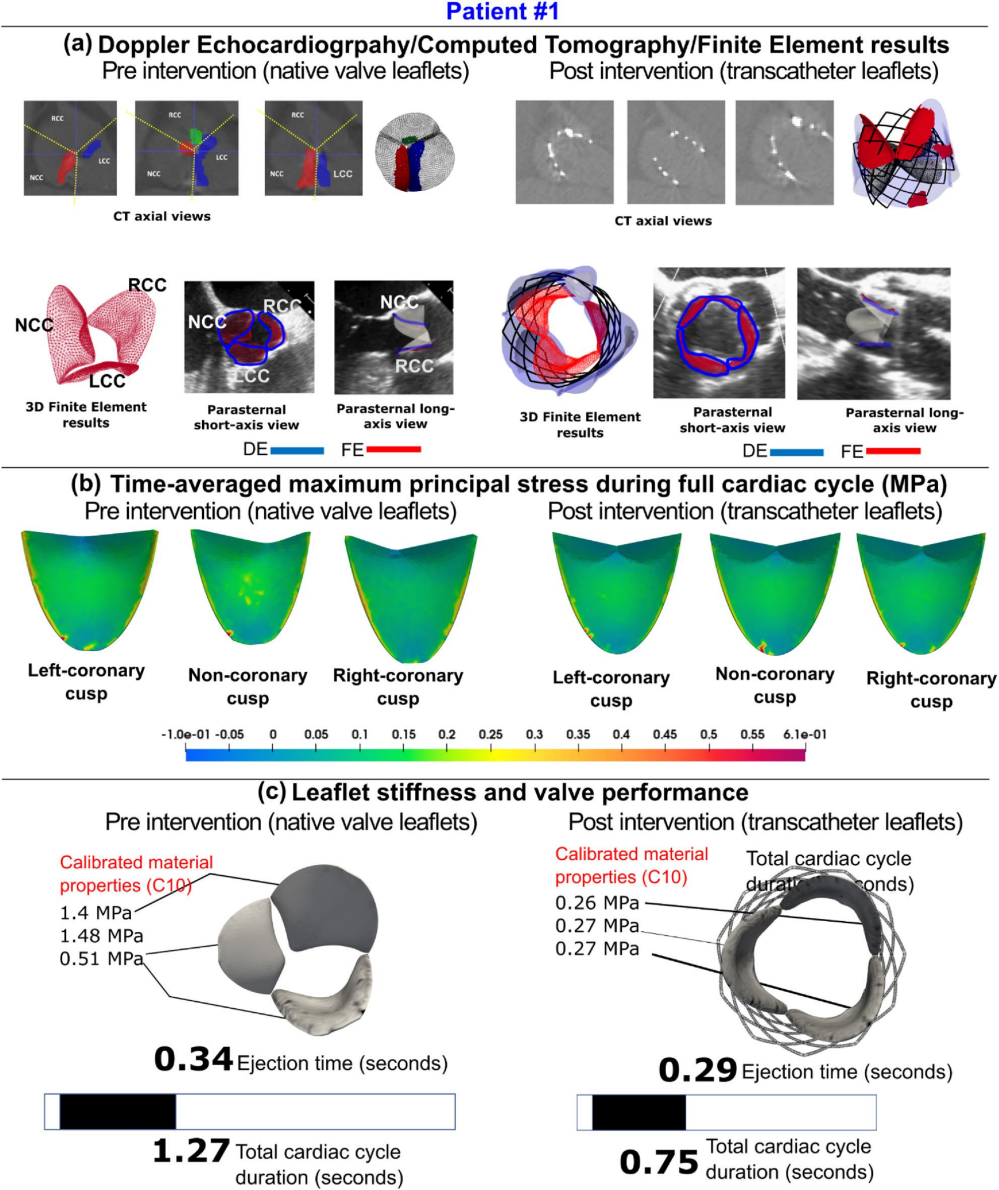
Comparison of Doppler-based finite element results with computed tomography and/leaflet-specific time-averages major principal stress stress/Doppler-based calibrated material properties- Patient #1. (**a**) Computed tomographic and Doppler echocardiographic images compared to the results of the Doppler-based finite element solver; (**b**) the time-averaged maximum principal stress on all native aortic valve leaflets pre-intervention and all transcatheter valve leaflets post-intervention; (**c**) results of the Doppler-based framework illustrating the material properties and leaflet stiffness as well as performance features such as ejection time and cardiac cycle duration.

**Figure 8:**
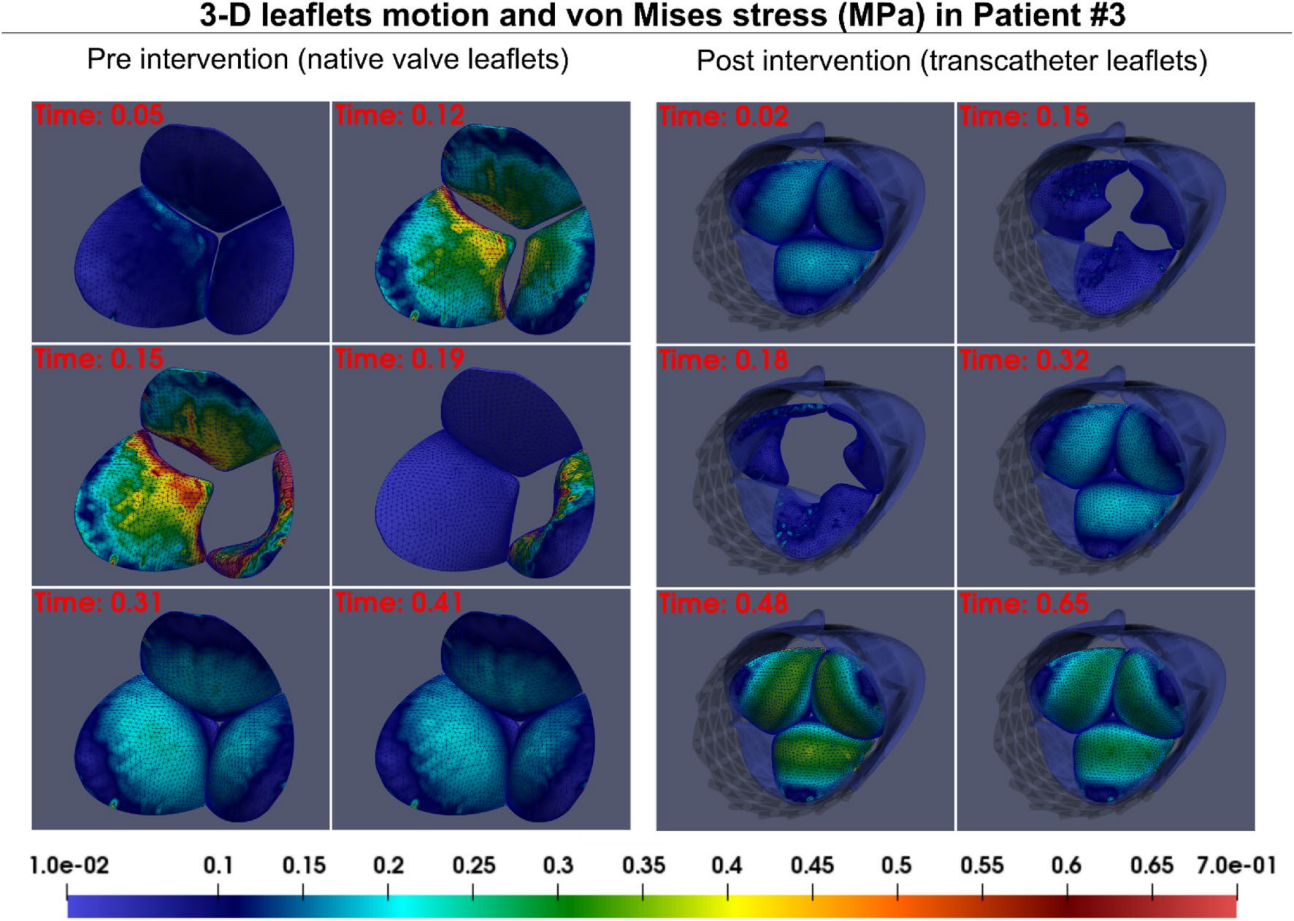
3D motion and 3D distribution contours of von Mises stress in patient #3 at six time points throughout the cardiac cycle in both pre- and post-intervention states. Using our framework, we estimated 3D deformation of aortic valve leaflet during full cardiac cycle as well as the von Mises stress distribution.

**Figure 9:**
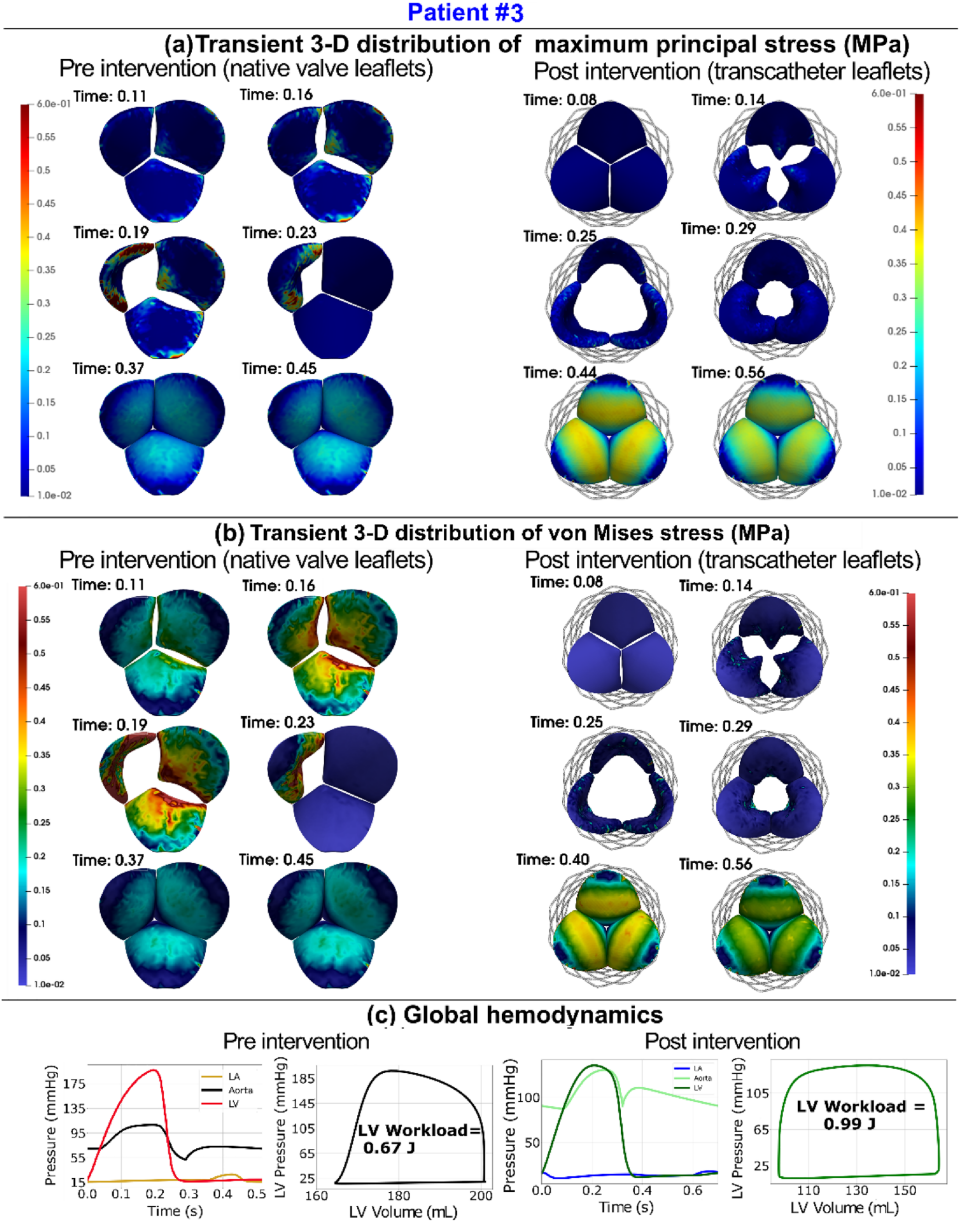
Changes in valve dynamics and global hemodynamics in patient #3 between baseline and 90-day post-TAVR. (**a**) Transient distribution of major principal stress at six time phases of the cardiac cycle: early systole (start of valve opening), early to peak systole (valve opening), peak systole (fully open), peak to late systole (valve closure), early diastole (closed configuration) and late diastole (closed configuration); (**b**) transient distribution of the von Mises stress at six time phases of the cardiac cycle: early systole(start of valve opening), early to peak systole(valve opening), peak systole (fully open), peak to late systole (valve closure), early diastole (closed configuration) and late diastole (closed configuration); (**c**) *Global hemodynamics*: LV workload; aorta and LV pressures in both pre- and post-intervention states. *Patient #3*: *Pre-TAVR*: severe aortic stenosis (EOA = 0.6 cm^2^), dyslipidemia, coronary artery disease, mild to moderate mitral regurgitation and chronic AF, ejection fraction: 29%, brachial pressures: 61 and 107 mmHg; *Post-TAVR*: aortic valve (EOA = 2.0 cm^2^), mild mitral regurgitation and dyslipidemia, ejection fraction: 34%, brachial pressures: 86 and 130 mmHg.

**Figure 10:**
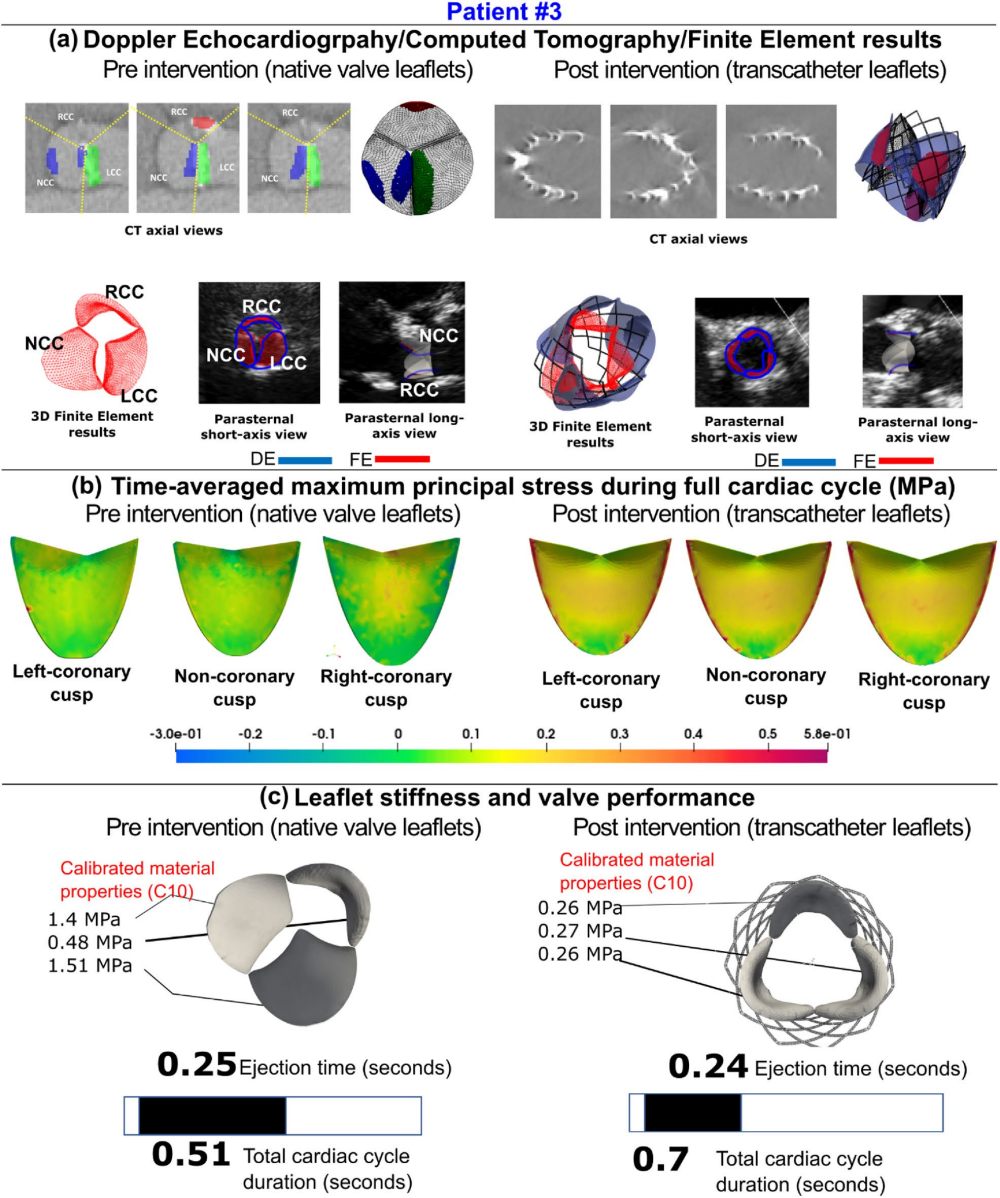
Comparison of Doppler-based finite element results with computed tomography and/leaflet-specific time-averages major principal stress/Doppler-based calibrated material properties-Patient #3. (**a**) Computed tomographic and Doppler echocardiographic images compared to the results of the Doppler-based finite element solver. (**b**) The time-averaged maximum principal stress on all native aortic valve leaflets pre-intervention and all transcatheter valve leaflets post-intervention. (**c**) Results of the Doppler-based framework illustrating the material properties and leaflet stiffness as well as performance features such as ejection time and cardiac cycle duration.

**Figure 11:**
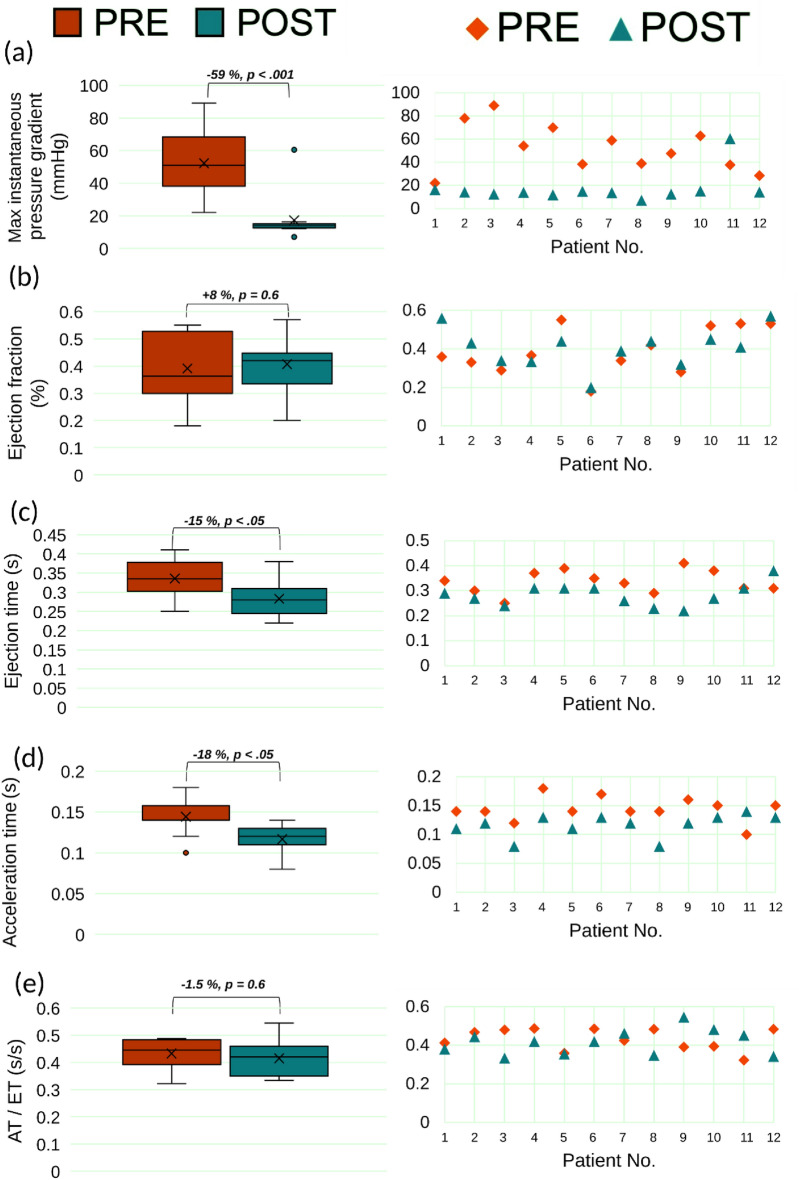
Clinical assessment of hemodynamics. Changes in clinical assessment of patients’ hemodynamics before and 90 days following TAVR (n = 12). (**a**–**e**) Column 1: Pre- and Post-TAVR hemodynamic measures (n = 12); (**a**) Maximum instantaneous Doppler pressure gradient (see Figures S7 and S8 for more details; Supplementary Material); (**b**) ejection fraction; (**c**) ejection time; (**d**) acceleration time, (**e**) ET/AT. Where statistical significance occurs, p values are indicated between paired variables in the box plot). Column 2: individual data points (n = 12) comparing pre- and post-TAVR measures for the same variable in column 1.

**Figure 12:**
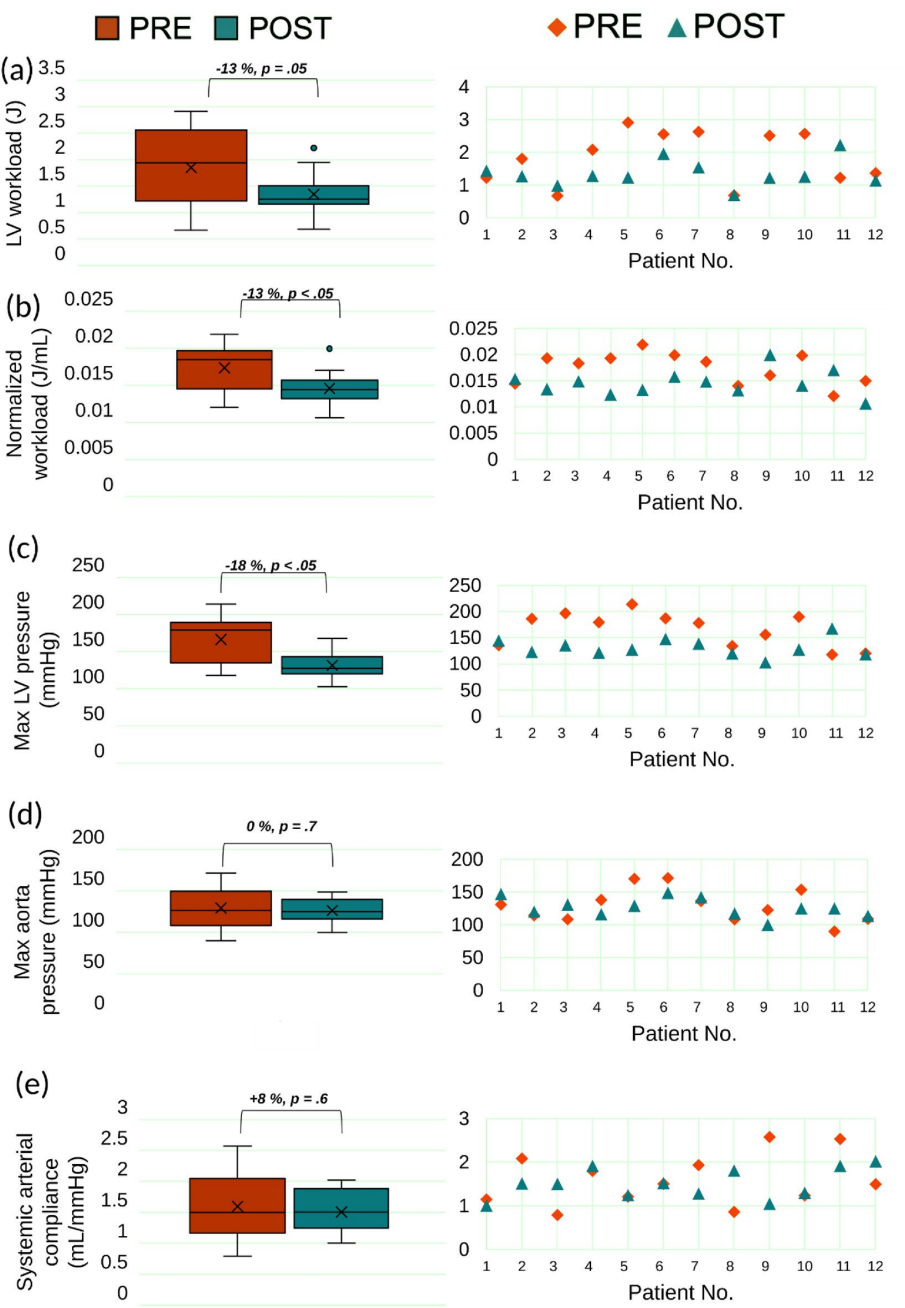
Global hemodynamics. Changes in predicted global hemodynamics before and 90 days following TAVR (n = 12). (**a**–**e**) Column 1: Pre- and Post-TAVR hemodynamic measures (n = 12); (**a**) LV workload; (**b**) normalized workload; (**c**) maximum left ventricle pressure; (**d**) maximum aorta pressure (see Figures S7 and S8 for more details; Supplementary Material); (**e**) systemic arterial compliance. Where statistical significance occurs, p values are indicated between paired variables in the box plot. Column 2: Individual data points (n = 12) comparing pre- and post-TAVR measures for the same variable in column 1.

**Figure 13:**
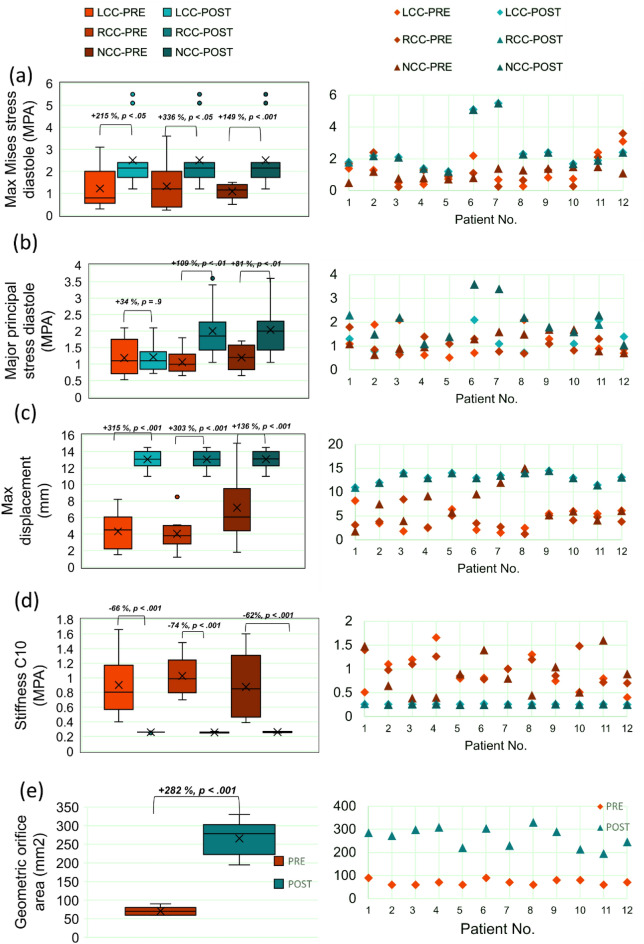
Valve dynamics. Changes in patients’ predicted valve dynamics before and 90 days following TAVR during the full cardiac cycle. (**a**–**e**) Column 1: Pre- and Post-TAVR hemodynamic measures (n = 12); (**a**) maximum von Mises stress at diastole; (**b**) major principal stress at diastole; (**c**) maximum displacement; (**d**) stiffness; (**e**) geometric orifice area. Where statistical significance occurs, p values are indicated between paired variables in the box plot for each leaflet. Column 2: individual data points (n = 12) comparing pre- and post-TAVR measures for the same variable in that column as Column 1.

We developed a Doppler-based computational mechanics diagnostic framework (Figs. 1, 2, 3; Table 1) to non-invasively investigate the dynamic behavior of the aortic valve (e.g., transient 3-D distribution of stress and displacement field, 3-D deformed shape of leaflets, geometric orifice area, angular positions of leaflets, stiffness, etc.; Figs. 4, 5, 6, 7, 8, 9, 10, 11, 12 and 13; Figures S1 to S8 (Supplementary Material)). This framework is based on a Doppler-based patient-specific lumped-parameter model (LPM)^38^, and a 3-D Doppler-based nonlinear (using anisotropic hyper elastic) finite element solver CalculiX^89^ (Figs. 1, 2: schematic diagrams; Fig. 3: algorithm flow chart). The designed LPM is an amalgamation of a parameter estimation algorithm and an a lumped-parameter module^38^ consisting of sub-models allowing for the analysis of all combinations of valvular, vascular and ventricular diseases (Fig. 1; schematic diagram). A Doppler-based parametric method was developed to construct the 3-D geometry of the asymmetric aortic valve leaflets. Calculations of this Doppler-based computational mechanics diagnostic framework was validated against clinical Doppler echocardiography data (Fig. 4) in 12 patients with AS (Table 1, Baseline patient characteristics). In this study, transthoracic echocardiogram (TTE) is utilized for the developments and transesophageal echocardiogram (TEE) is used exclusively for validation.

**Table 1:** Baseline patient characteristics including patient description, arterial hemodynamics, aortic valve hemodynamics and left ventricle hemodynamics.

	AS patients (n = 12, mean ± SD)
Patient description	
Mean age (years)	76 ± 3.5
Gender	(Male: 9; female: 3)
Mean weight (kg)	69 ± 12.5
Mean height (cm)	169.9 ± 10.6
Body surface area (m^2^)	1.7 ± 0.11
Body mass index (kg/m^2^)	32.1 ± 24.8
Arterial hemodynamics	
Systolic arterial pressure (mmHg)	Pre-TAVR: 131.16 ± 26.13; post-TAVR: 124.9 ± 16.25
Diastolic arterial pressure (mmHg)	Pre-TAVR: 61.16 ± 10.46; post-TAVR: 61.0 ± 12.09
Aortic valve hemodynamics	
Stenotic aortic valve effective orifice area (cm^2^)	0.725 ± 0.135
Stenotic aortic valve type	Tricuspid: 12; Bicuspid: 0
Prosthetic size (mm)	25 ± 2.83
Prosthetic type	Edwards SAPIEN (n = 12)
Maximum aortic valve pressure gradient (mmHg)	Pre-TAVR: 52.22 ± 20.37; post-TAVR: 17.26 ± 13.8
Mean aortic valve pressure gradient (mmHg)	Pre-TAVR: 29 ± 18.6; post-TAVR: 12.5 ± 4.7
Left ventricle hemodynamics	
Ejection fraction (%)	Pre-TAVR: 39 ± 11; post-TAVR: 41 ± 1
Heart rate (bpm)	Pre-TAVR: 70 ± 25.7; post-TAVR: 70.52 ± 10.56

### Clinical medical imaging

#### Study population and data acquisition

Our study selected 12 patients retrospectively with severe AS who had undergone TAVR at St. Paul’s Hospital (Vancouver, Canada). The medical records of these patients provided demographic and procedural data (see Table 1 for details). The de-identified and anonymized data was transferred from St. Paul’s Hospital and the approval was granted by the Clinical Research Ethics Board (CREB) and informed consents were collected from all participants. Only patients who underwent a full range of imaging procedures including transthoracic and transesophageal echocardiograms, as well as computed tomography (CT) imaging for the assessment of calcification, were included in the study. All methods and measurements were performed in accordance with the relevant guidelines and regulations including guidelines of the American College of Cardiology and American Heart Association. Results derived from the findings of the study were expressed as mean ± standard deviations (SD).

#### Doppler echocardiography

Doppler echocardiography (DE) data included raw images and documented reports, and were collected at the baseline, and at 90-day post procedure. Echocardiograms and reports were reviewed and analyzed by senior cardiologists using OsiriX imaging software (OsiriX version 8.0.2; Pixmeo, Switzerland). The following metrics were measured:

##### Input parameters of the LPM

The following input parameters were utilized by the model: forward left ventricular outflow tract stroke volume, heart rate, ejection time, ascending aorta area, left ventricular outflow tract area, aortic valve effective orifice area, mitral valve effective orifice area, and grading of aortic and mitral valves regurgitation severity. Each of the aforementioned parameters can be reliably obtained using DE imaging^38^ (see Fig. 1; Table 1 for details).

##### Patient-specific Doppler-based 3-D geometry of aortic valve leaflets

Recently, parametric models of aortic valve leaflets have been shown to realistically represent the function of the aortic valve^82,90^. The method applied in this study was originally proposed by Thubrikar^91^, further developed by Labrosse^92^ and later modified by Morganti^68,93^, who removed the symmetric simplifying assumption (see Fig. 2b,c). The parametric model was implemented by means of the 3-D CAD software Autodesk Inventor 2017^94^ to construct the aortic valve leaflet geometry of patients for both pre- and post-TAVR using 2-D DE images (parasternal long-axis and short-axis views, Fig. 2). For this purpose, the essential parameters were measured using DE images and are as follows: base diameter (${\mathrm{D}}_{\mathrm{b}}$), diameter of commissures (${\mathrm{D}}_{\mathrm{c}}$), valve height ($\mathrm{H}$), length of coaptation (${\mathrm{X}}_{\mathrm{s}}$) and two angles of the leaflets (α and β); all measured at the end of diastole:

1. As depicted in Fig. 2a the conical frustrum is comprised of three essential dimensions, the diameter of the base (${\mathrm{D}}_{\mathrm{b}})$, the diameter of commissures (${\mathrm{D}}_{\mathrm{c}}$) and the valve height ($\mathrm{H}$). These dimensions were measured on the long-axis parasternal Doppler echocardiography plane view (see Fig. 2a). The conical frustum is composed of three surfaces: two circular flat surfaces (shown in blue and red hatch in Fig. 2c (step 1) and a curved surface. The plane M (shown with pink lines in Fig. 2c (steps 1, 4 and 5)) is passing through the upper surface (shown in red hatch in Fig. 2c (step 1)).
2. To consider the asymmetry of the leaflets, two angles, α and β were measured via the parasternal short-axis view to mark three points (A,B and, C) on the base of the conical frustrum (Fig. 2c, steps 2 and 3). Three additional points (A′, B′ and C′) with a rotation of 180° were also marked on the upper circle of the conical frustum (Fig. 2c, steps 2 and 3).
3. For each leaflet, a plane was derived by three points, two on the upper surface with another one on the base surface (Fig. 2c, step 3). A trio of intersection curves were defined as the intersection between the cone-shaped surface and the three planes (Fig. 2c, step 4). The first intersection curve (1) was defined between the first plane (1) and the cone-shaped surface (Fig. 2c, step 3). Each of the subsequent curves was created between the numbered plane and the cone-shaped surface (Fig. 2c, steps 3 and 4).
4. The curves represented in green depict the attachment between the leaflets and the aortic root (Fig. 2c, step 4). All green curves were projected to plane M (Fig. 2c, Step 5), and their projection axes were measured to be 5.25° from the Z axis (Fig. 2c, supplement 1). The open configuration of the leaflets was defined by the projected curves on plane M (Fig. 2c, step 5; Fig. 2c, supplement 2). Each leaflet was defined by an intersection curve (shown in green), its projection on plane M (shown in purple), and the marched surface of the loft technique (Fig. 2c, step 5).
5. Employing the Thubrikar approach using the symmetry planes, an approximation of the closed configuration of the leaflets was derived. To achieve this, each leaflet in its fully open configuration was mirrored by its corresponding plane marked in orange lines (Fig. 2c, step 5 and supplement 2). Furthermore, the intersection region that resulted from the closed configuration was removed from each leaflet (see the hatched area in Fig. 2c, supplement 2).
6. Using parameter ${X}_{S}$, the coaptation area was added to the closed configuration of each leaflet (Fig. 2a). ${X}_{S}$ represents the length of the coaptation region for each leaflet in the Z direction. Three additional points, X, Y and, Z were defined with the ${X}_{s}$ distance on the top of the free edge depicted in blue (Fig. 2c, step 6).Two lines were then used to connect points X and Y as well as points Y and Z, both displayed in red (Fig. 2c, supplement 3). Finally, the blue edges were extended to the red lines in the Z direction (see Fig. 2c, supplement 3).

### Patient-specific Doppler-based finite element modeling

In this study, the dynamic behaviour of the aortic valve leaflets was modelled using motion equations solved by a non-linear finite element method. Previous studies have been conducted with several limitations to address the various challenges associated with solving motion equations of aortic valve leaflets. The limitations are as follows: (1) motion equations with quasi-static assumptions were commonly applied^75,83,85^; (2) isotropic hyper-elastic models were used, though lack the ability to consider the anisotropic structure of the aortic valves leaflets^13,27,68,70,71,73,77,82,84,95,96^; (3) often there were constant or non-patient specific boundary conditions and pressure loads applied to the models; (4) material properties were not calibrated for each individual patient^2,13,27,66,67,69,71–73,76,78–80,82–85,88^; and (5) symmetric configuration of leaflets were assumed^27,67,78–81,84,86,88^. Multiple methods were applied in this study to overcome these limitations including a finite element simulation that was performed by addressing the non-linearities of the aortic valve leaflet motion. In addition, a fibre-reinforced constitute law was used, representing the anisotropic structure of aortic valve leaflets. In terms of boundary conditions, patient-specific transient pressure loads were calculated using our previously developed and validated LPM^38^. The hyper-elastic material properties of each leaflet were calibrated using parasternal long and short axis DE plane views for each patient. Finally, the asymmetric configuration of aortic valve leaflets was considered in 3-D geometry construction of leaflets using DE images.

#### Finite element solver

To define the dynamic equation of motion, the principals of conservation of mass and linear momentum must be considered in conjunction and can be expressed as follows:

1 $${\rho }_{\mathrm{s}}\frac{{\mathrm{D}}^{2}\mathrm{U}}{{\mathrm{D}}^{2}{\mathrm{t}}^{2}}=\nabla .\mathrm{P}+{\rho }_{\mathrm{s}}\mathrm{f}$$

where $P$, $f$, ${\rho }_{s}$, $U$ and $\frac{{D}^{2}(U)}{{D}^{2}{t}^{2}}$ are the Piola transform of the Cauchy stress tensor (viz. Piola–Kirchhoff stress tensor), the body force (e.g., unit weight of the material), the density, the displacement and the second rate operator, respectively. To complement Eq. (1), a hyper-elastic constitutive law characterizing the mechanical behavior of aortic valve leaflets is taken into consideration:

2 $$\mathrm{P}=\mathrm{C}:\mathrm{E}$$

3 $$\mathrm{E}=\frac{1}{2}\left({\mathrm{F}}^{\mathrm{T}}\mathrm{F}-\delta \right)$$

where $C$, $E$ and $F$ are the elasticity tensor, the Green–Lagrange strain tensor, and the deformation gradient (the unit tensor), respectively. To calibrate the stiffness tensor ($C$), performing experimental tests are necessary which can be found in the Material properties section.

As complex geometries and nonlinearities are present in the aortic valve leaflets, the governing equation of motion (Eq. 1) cannot be solved analytically. To solve Eq. (1), its weak (variational) form employing the Galerkin method and incorporating finite element discretization is applied^89,97^. By doing so, the semi-discrete form of the governing equations is obtained as follows:

4 $$[\mathrm{K}]\{\mathrm{U}\}+[\mathrm{M}]\frac{{\mathrm{D}}^{2}}{{\mathrm{Dt}}^{2}}\{\mathrm{U}\}=\{\mathrm{F}\}$$

where $[K]$ is the global stiffness matrix, $[M]$ represents the global mass matrix, and $\{F\}$ denotes the global force vector. Euler time-discretization of motion equations was applied using the implicit $\alpha$-method of Miranda et al.^98^. The time steps were kept small enough (1E−4) to prevent divergence of the nonlinear Newton solver .SPOOLES library was used using a multi-threaded approach to solve linear equations^99^. CalculiX (version 2.15, an open-source package) was applied for the dynamic finite element simulation^89^.

#### Boundary condition

When analyzing local flow dynamics, it is imperative to apply the patient-specific boundary condition as both upstream and downstream conditions can alter the obtained results. Each leaflet is comprised of two surfaces, one on the ventricular side and another on the side of the aorta. Initially, the faces connected to the root have zero displacement as depicted in Fig. 1. The time-dependent pressure boundary conditions (both aorta and LV side) were calculated using our patient-specific Doppler-based lumped-parameter model and were imposed on both surfaces of the leaflets (see Fig. 1). We should emphasize that the pressure difference between the LV and aorta can define the dynamic behavior of the aortic valve, not each of these pressures independently. We applied the dry assumption (devoid of flow) in our finite element simulation as validated by Van de vosse et al.^100^, who performed a fluid–structure simulation to analyze pressure distribution around the aortic valve. Their results confirmed that the time-dependent pressure imposed on the leaflet surfaces is nearly uniform across the entire leaflet. It should be noted that non-uniform pressure loads can be estimated using fully-coupled fluid–structure interaction methods, although at a higher computational cost compared with pure structural analysis^101,102^. In general, the dynamic behavior of the valve leaflets in these models is slightly different from purely structural methods based on uniform pressure loads^103,104^. In terms of kinematic parameters such as ejection time, rapid valve opening time, and rapid valve closing time, it has been shown that the results of fluid–structure interaction (FSI) simulation based on non-uniform pressure loads and purely structural simulation based on uniform pressure loads differ by an order of milliseconds^103,105^. Also in terms of stress distribution, structure simulation and fluid–structure interaction simulation provide similar results^103^. However, due to complex blood flow patterns and vortex shedding around the free edges, there are small fluctuations in stress values (leaflet fluttering phenomenon)^101,106,107^. To consider the effect of the surrounding fluid, we employed the viscous damping effects in our computational framework using the coefficients suggested by Marom et al.^108^. All aortic valve geometries were reconstructed using DE images taken at late diastole to obtain the best possible unpressurized geometry. All simulations were performed during the entirety of the cardiac cycle.

#### Material properties

Experimentally, it has been demonstrated that the stiffness of the aortic-valve leaflets in the circumferential direction can be on the order of six to eight times greater than that of the radial direction. The difference can be linked to the collagen fibers which are aligned in the circumferential configuration thereby creating a stiffer behaviour in that direction^91^. Despite the fact that several experimental studies demonstrated that the aortic valve leaflets are anisotropic^109–111^, some finite element studies focused on the importance of anisotropic behavior of aortic valve leaflets^112^. For instance, Zakerzadeh et al.^33,46^ showed that in small strain movements of aortic valve leaflets (closure time), assuming isotropy has considerable effects on the finite element results and particularly with regards to the stress distribution. In another study, which used rotation-free shell elements, it was shown that using isotropic behavior can impact the deformed leaflet configuration and underestimate displacements during valve closure^46^. Shifts in the location of peak leaflet stress were found in a comparative study focused on the effect of anisotropy of leaflets on finite element results which contradicts the implications of the isotropic assumption^26^. Furthermore, with regards to the effect of blood flow on the dynamic behavior of leaflets, compared with isotropic leaflets, anisotropic leaflets showed less fluttering during systole^113^. In this study, we employed the following strain energy function that is composed of isotropic and anisotropic terms^114^:

5 $$\mathrm{U}={\mathrm{U}}_{\mathrm{iso}}+{\mathrm{U}}_{\mathrm{aniso}}={\mathrm{C}}_{10}({\overline{\mathrm{I}} }_{1}-3)+\frac{1}{{\mathrm{D}}_{1}}{(\mathrm{J}-1)}^{2}+\sum_{\mathrm{i}=0}^{\mathrm{n}}\frac{{\mathrm{k}}_{1\mathrm{i}}}{2{\mathrm{k}}_{2\mathrm{i}}}[{\mathrm{e}}^{{\mathrm{k}}_{2\mathrm{i}}{({\mathrm{I}}_{4\mathrm{i}}-1)}^{2}}-1]$$

where ${\mathrm{U}}_{\mathrm{iso}}$ and ${\mathrm{U}}_{\mathrm{aniso}}$ are isotropic and anisotropic strain energy functions, respectively. ${\overline{\mathrm{I}} }_{1}$ and $\mathrm{J}$ are the first strain invariant and volumetric expansion ratio, respectively.${ C}_{10}$ and ${D}_{1}$ are material constants for the isotropic neo-Hookean material model for the matrix. Incompressibility of the aortic valve tissue^115^ forces the second term of the isotropic component ($\frac{1}{{\mathrm{D}}_{1}}{(\mathrm{J}-1)}^{2}$) to zero. To avoid divergence, we assume a conservative incompressibility value (i.e., a Poisson's ratio of 0.475) in our simulations. In several studies, the assumption of nearly compressible behaviour was used to simulate cardiac tissues using finite elements^115–118^.

The isotropic matrix is reinforced by exponential strengthening terms (${\mathrm{U}}_{\mathrm{aniso}})$ in the circumferential fiber direction. Parameter $n$ defines the number of fiber family direction with the assumption that the circumferential direction ($n$ =1) was the dominant alignment of fibers^119,120^. For each leaflet, a local cylindrical coordinate system was implemented in the finite-element solver, wherein the fiber orientations of each tetrahedral element are assigned^108^. Constants,${k}_{1}$ and ${k}_{2}$,denote the required values for describing the anisotropic component and thus, there are three material constants (${C}_{10},{k}_{1}$ and ${k}_{2}$) to be determined. As the nature of the anisotropic component of Eq. (5) is exponential, an incorrect initial estimation while running the finite-element code will result in divergence. A sub-iteration was therefore implemented to avoid any divergence during numerical iterations of the Newton–Raphson method for each time step.

In this study the constitutive model of Eq. (5) was used for aortic-valve leaflets. However, more complicated constitutive equations can be used for aortic valve leaflets. For instance Eq. (5) can be modified to consider fiber dispersions^121^. More sophisticated methods to account for the local alignment of collagen fibers of each aortic valve leaflet have also been proposed^80,108,114^. Kim^114^ used beam and shell elements to represent collagen fibers and the elastin matrix, respectively. Marom et al.^80,119^ have adopted a similar method for an FSI simulation of a bicuspid aortic valve. They applied the method to porcine aortic valve by obtaining the fiber orientations of leaflets using microscopy. Although currently this is the most detailed model, its usage for clinical applications is unpractical with the limitations of current non-invasive imaging modalities to obtain patient-specific fiber orientation of the aortic valve leaflets^122^. To decrease the number of unknown parameters of the anisotropic constitutive model of the aortic valve tissues, Eq. (5) was used in this study.

Although several studies applied porcine material properties for human, Martin et al. demonstrated that human aortic valve tissue was stiffer (in the radial and circumferential direction) than that of the porcine counterpart^123^. Moreover, any solution to estimate material properties for clinical applications should be practical and based on non-invasive clinical data.

Detailed information on the mechanical properties of calcified regions of aortic valve leaflets, especially for human, is not currently available. It was observed that calcified regions are more brittle than healthy leaflets^91^ and therefore, two simplifying methods may be used to model calcification:

1. Increasing the stiffness of the leaflets^120^ to capture geometrical parameters such as effective orifice area (EOA), geometric orifice area (GOA) or angular position. Calcified leaflets are less elastic than healthy ones, therefore their deformation is restricted. As leaflet stiffness increases, they become more resistant to deformation, resulting in a lower geometric orifice area, which is the area available during systole^124^. Healthy leaflets, on the other hand, with greater elastic material qualities, give more area for blood flow, resulting in a larger geometric orifice area^125^. It is worth noting that the dynamic behaviour of leaflets, such as time-dependent deformation, is also influenced by pressure loads and the geometry of the aortic valve^122^.
2. Modifying local thickness or material properties using CT images of calcified regions of aortic valve leaflets. An increase in thickness or stiffness can reproduce calcification effects on specific regions of leaflets^114,123^.

In this study, the first method was applied to stiffen each calcified leaflet assuming that calcification only affects the isotropic component of the leaflets^67^. Since the tissue is incompressible, only one constant ${(C}_{10})$ (Eq. 5) must be determined to model calcification. We avoided applying a single set of parameters to all leaflets but rather determined the material properties of each leaflet separately. To perform this, DE plane views were reconstructed in the computational domain to directly compare geometrical parameters such as angular position or visible area of leaflets with DE images. The DE parasternal short-axis plane view was used to calibrate material properties of the left coronary leaflet and DE parasternal long-axis plane views was used to calibrate non-coronary and right-coronary leaflets material properties (see Fig. 3).

#### Mesh study

All 3-D geometries reconstructed using DE images (“Patient-specific Doppler-based 3-D geometry of aortic valve leaflets”) were discretized into second-order tetrahedral elements^89^. Mesh generation was performed in Gmsh open-source package^126,127^. Each case consisted of a unique number of mesh elements as a result of asymmetrical geometry and differing geometric input parameters. In our simulation, the largest element edge size was always less than 0.62 mm and the total number of elements for all 24 cases investigated in this study (12 patients) ranged between 26,000 and 37,000 tetrahedral elements. All meshes are generated using quadratic tetrahedral elements (C3D10). The elements have four points at each tetrahedron vertex and six points in the middle of each of their six edges. In accordance with prior literature, quadratic (second-order) elements were used as they aid in avoiding numerical locking^89,128,129^. Similar element choice (one quadratic element though the thickness) for thin cardiac structure like aortic valve leaflet or mitral leaflets have been used in several studies^105,130–133^.To assess mesh convergence, mesh definition for a solid domain was deemed acceptable when variation of less than 1% (insignificant) were found in the principal stress, the von Mises stress and the maximum leaflet displacement (for each leaflet) in successive meshes^133^. Moreover, time step independency was studied for all models in which the differences in results (e.g., major principal stress, von Mises stress as well as the maximum displacement) were not significant (less than 0.5%)^133^. The solution marched in time with a time step of 10^–4^ s for all 12 patients. Finally, convergence was obtained when the sum of all residuals reached a value lower than 10^–8^.

#### Sensitivity analyses

Due to the complex multi-physics nature of the heart valves, the overall estimation of valve biomechanical parameters is very dependent on the outputs of the lumped-parameter model. Our patient-specific Doppler-based lumped-parameter model, which provided boundary conditions, was validated against clinical catheterization data in 49 C3VD patients with a substantial inter- and intra-patient variability with a wide range of disease^38^. In the present study, we used the validated lumped-parameter model^38^ to obtain time varying left ventricle and aorta pressures as the inputs to the solid model of the aortic valve. To determine the cardiac parameters, we analyzed parameter sensitivity of the outputs used in our present study derived from the lumped-parameter model^134^. We found that the outputs from the lumped-parameter model were increasingly sensitive to the forward left ventricular outflow tract stroke volume (forward LVOT-SV, an input parameter to the lumped-parameter model): LV pressure: 27%, LV Volume 19% by a ± 20% change in the Forward LVOT-SV. Though other inputs were analyzed, none significantly affected the output values. We should address that Forward LVOT-SV is measured reliably using Doppler echocardiography with high accuracy and sensitivity of the model to this parameter does not jeopardize the obtained results. In addition, sensitivity analysis revealed negligible effects of changes (± 20%) in the free parameters on the model output variables. As depicted in Fig. 4, the results obtained with Doppler-based 3-D non-linear finite element solver and lumped-parameter model were validated against clinical TEE data in patients. Our results showed positive agreement of the angular position calculated utilizing the computational framework with that obtained through TEE data in 12 patients investigated in the study.

The time-dependent pressure boundary conditions (both aorta and LV side) were calculated using our patient-specific lumped-parameter model and were imposed on the surface of the leaflets of both aorta and left ventricle (LV) sides. The pressure difference existing between the LV and the aorta are capable of defining the dynamic behaviour of the aortic valve. It should be noted the dynamic behaviour cannot be expressed by the individual pressures measured from the aorta or LV. To study the sensitivity of the Doppler-based finite element results (the second step) to the results obtained using the Doppler-based lumped-parameter model (the first step), ± 10% variations in the aorta pressure (both peak and minimum pressures) and the maximum pressure gradient across the aortic valve during the cardiac cycle (pressure difference between the LV and aorta) were considered. The resulting peak von Mises stress, average von Mises stress, and calibrated material properties of the sensitivity analysis were tabulated and presented in Table 2.We observed that the maximum pressure gradient during the cardiac cycle across the aortic valve is the most determinant parameter and showed up to 7% change in the results due to ± 10% variations as described above (Table 2). The results were less sensitive to aorta maximum pressure, and the least sensitive to aorta minimum pressure. Be advised that the ± 10% artificial variations that were defined in the lumped-parameters results can be related to variations in the patient condition/pathology if they were to be measured in the real patient. These artificial fluctuations are important as they should have some effect on the finite-element analysis results. We also investigated the sensitivity of the calculated angular rotation as well as the leaflets’ area to the slight changes (± 5% variations) in the estimated material properties. We observed that the calculated angular rotation as well as leaflets’ area showed up to 4% and 3% changes, respectively.

**Table 2:** The changes in computed calibrated material properties (C10), average von Mises stress, and peak von Mises stress due to ± 10% variations in the aorta pressure (both peak and minimum pressures) and the maximum pressure gradient across the aortic valve during the cardiac cycle (pressure difference between the LV and aorta).

	Left coronary leaflet			Right coronary leaflet			Non-coronary leaflet			Left coronary leaflet			Right coronary leaflet			Non-coronary leaflet		
	C10	Mean von Mises	Max von Mises	C10	Mean von Mises	Max von Mises	C10	Mean von Mises	Max von Mises	C10	Max von Mises	Max Mises	C10	Mean von Mises	Max von Mises	C10	Mean von Mises	Max von Mises
	10% change in maximum aorta pressure									-10% change in maximum aorta pressure								
Patient #10	− 2.80%	− 2.40%	− 3.40%	− 3.65%	− 2.60%	− 3.20%	− 3.65%	− 2.53%	− 3.40%	4.10%	4.10%	4.90%	4.20%	3.95%	4.90%	4.00%	4.00%	4.90%
Patient #9	− 2.40%	− 2.80%	− 2.90%	− 3.10%	− 2.10%	− 3.70%	− 2.90%	− 2.45%	− 3.00%	2.84%	3.40%	4.45%	3.66%	3.41%	4.10%	3.40%	3.45%	4.11%
	+ 10% change in minimum aorta pressure(mmHg)									− 10% change in minimum aorta pressure(mmHg)								
Patient #10	0.00%	− 0.40%	− 0.10%	0.00%	− 0.35%	− 0.20%	0.00%	− 0.35%	0.00%	0.00%	0.60%	0.00%	0.00%	0.44%	0.00%	0.00%	0.45%	0.00%
Patient #9	0.00%	− 1.00%	− 0.20%	0.00%	− 0.40%	− 0.20%	0.00%	− 0.25%	0.00%	0.00%	0.40%	0.00%	0.00%	0.19%	0.00%	0.00%	0.60%	0.00%
	+ 10% change in maximum pressure gradient across the aortic valve (pressure difference between the left ventricle and aorta)									− 10% change in maximum pressure gradient across the aortic valve (pressure difference between the left ventricle and aorta)								
Patient #10	5.80%	5.80%	7.13%	6.10%	5.70%	5.95%	5.70%	6.30%	5.80%	− 5.10%	− 4.30%	− 3.90%	− 5.40%	− 4.30%	− 4.10%	− 5.00%	− 4.40%	− 4.40%
Patient #9	5.42%	4.90%	6.40%	7.10%	6.00%	6.70%	4.90%	6.10%	6.20%	− 4.70%	− 4.10%	− 6.80%	− 4.49%	− 5.10%	− 6.90%	− 3.90%	− 3.80%	− 7.00%

Finally, it is essential to evaluate the framework's sensitivity analysis against potential variations brought on by image noise, image quality, and observer variability^128,129,135^. To study the sensitivity of the Doppler-based finite element results versus the six-dimensions measured using Doppler echocardiography images, ± 5% variations were considered^136,137^. The calibrated material properties, average von Mises stress, and peak von Mises stress were calculated over a cardiac cycle (see Table 3 for a sample patient). The maximum von Mises stress showed the greatest changes (up to 8%) to the variations in geometrical parameters. Compared to the maximum von Mises stress, the average von Mises stress was less sensitive to the changes in geometrical parameters (up to 4%). Additionally, the most important geometrical parameters are the valve height and base diameter, and variations of these parameters will result in more variations of the finite element results. From the geometrical parameters, the coaptation length was the least effective one on the finite element results. Finally, it should be concluded all the measured dimension have sensible effects on the finite element results. We had similar observations for the other patients investigated in this study.

**Table 3:** The changes in computed calibrated material properties (C10), mean von Mises stress, and maximum von Mises stress due to ± 5% variations in the six measured parameters from Doppler echocardiography (TTE) for a sample patient.

	Left coronary cusp			Right coronary cusp			Non-coronary cusp			Left coronary cusp			Right coronary cusp			Non-coronary cusp		
	C10	Mean von Mises	Max Mises	C10	Mean von Mises	Max Mises	C10	Mean von Mises	Max Mises	C10	Mean von Mises	Max Mises	C10	Mean von Mises	Max Mises	C10	Mean von Mises	Max Mises
	+ 5% change in diameter of base (${D}_{b}$ )									-5% change in diameter of base (${D}_{b}$)								
Patient #11(preTAVR)	2.00%	− 4.00%	− 7.30%	1.80%	− 4.40%	− 6.80%	1.40%	− 3.40%	− 6.70%	− 1.40%	5.10%	8.10%	− 2.78%	2.80%	7.50%	− 1.11%	2.80%	8.10%
Patient #11(postTAVR)	1.10%	− 2.70%	− 6.10%	1.10%	− 2.70%	− 6.10%	1.10%	− 2.70%	− 6.10%	− 2.50%	3.44%	7.50%	− 2.50%	3.44%	7.50%	− 2.50%	3.44%	7.50%
	+ 5% change in diameter of commissures (${D}_{c}$)									− 5% change in diameter of commissures (${D}_{c}$)								
Patient #11(preTAVR)	− 1.40%	2.70%	3.90%	− 2.10%	1.22%	3.10%	− 1.40%	1.55%	3.55%	1.00%	− 2.22%	− 4.44%	1.88%	− 3.44%	− 6.90%	2.00%	− 2.80%	− 4.50%
Patient #11(postTAVR)	− 2.77%	1.40%	3.10%	− 2.77%	1.40%	3.10%	− 2.77%	1.40%	3.10%	1.40%	− 1.40%	− 2.69%	1.40%	− 1.40%	-2.69%	1.40%	− 1.40%	− 2.69%
	+ 5% change in valve height ($H$)									− 5% change in valve height ($H$)								
Patient #11(preTAVR)	− 1.02%	3.10%	8.80%	− 0.95%	2.40%	6.10%	− 1.33%	2.90%	7.77%	0.99%	− 2.40%	− 6.22%	1.70%	− 2.55%	− 8.50%	1.22%	− 3.50%	− 8.70%
Patient #11(postTAVR)	− 0.79%	2.10%	5.89%	− 0.79%	2.10%	5.89%	− 0.79%	2.10%	5.89%	1.11%	− 1.40%	− 5.40%	1.11%	− 1.40%	− 5.40%	1.11%	− 3.40%	− 5.40%
	+ 5% change in Coaptation length (${X}_{s}$ )									− 5% change in Coaptation length (${X}_{s}$ )								
Patient #11(preTAVR)	0.20%	− 1.40%	− 2.77%	0.10%	− 2.00%	− 3.50%	0.10%	− 0.80%	− 2.40%	0.10%	2.44%	2.40%	0.40%	1.80%	3.40%	0.77%	0.48%	2.50%
Patient #11(postTAVR)	0.00%	− 2.00%	− 3.10%	0.00%	− 2.00%	− 3.10%	0.00%	− 2.00%	− 3.10%	0.00%	1.99%	3.90%	0.00%	1.99%	3.90%	0.00%	1.99%	3.90%
	+ 5% change in asymmetry angle ($\alpha$)									− 5% change in asymmetry angle ($\alpha$)								
Patient #11(preTAVR)	3.00%	− 2.99%	− 7.88%	− 2.50%	3.55%	3.88%	0.00%	0.00%	0.00%	− 2.44%	3.77%	4.11%	− 2.10%	2.40%	6.10%	0.00%	0.00%	0.00%
Patient #11(postTAVR)	2.00%	− 1.44%	− 4.11%	− 1.89%	4.10%	8.20%	0.00%	0.00%	0.00%	− 1.34%	2.89%	5.30%	− 3.10%	2.88%	4.89%	0.00%	0.00%	0.00%
	+ 5% change in asymmetry angle ($\beta$)									− 5% change in asymmetry angle ($\beta$ )								
Patient #11(preTAVR)	− 2.35%	3.27%	4.30%	0.00%	0.00%	0.00%	− 2.10%	2.40%	6.10%	3.00%	− 2.99%	− 7.88%	0.00%	0.00%	0.00%	− 2.30%	3.05%	4.66%
Patient #11(postTAVR)	− 1.65%	2.70%	5.55%	0.00%	0.00%	0.00%	− 3.10%	2.88%	4.89%	2.00%	− 1.44%	− 4.11%	0.00%	0.00%	0.00%	− 1.50%	3.10%	8.20%

#### Numerical simulation strategy

Figure 3a demonstrates the role of each individual part of the framework including the LPM method, the 3-D image reconstruction module, and the finite element solver. The framework initially processed parasternal long and short-axis DE views to reconstruct the leaflets. Subsequently the boundary conditions are calculated by patient-specific LPM and discretized geometry produced by Gmsh were implemented in CalculiX, as the non-linear finite element solver. Using the geometrical parameters (angular positions and GOA) measured at peak systole (fully open configuration of aortic valve) the material calibration is performed. For material calibration of non-coronary cusp (NCC) and right-coronary cusp (RCC), peak-systole time frame of parasternal long-axis view from TTE images was used. As the left-coronary cusp (LCC) is not visible in the parasternal long-axis view, the parasternal short-axis view is employed. The sub-iterations and details are as follows:

1. The right coronary cusp (RCC) and non-coronary cusp (NCC) are highlighted in parasternal long axis echocardiography plane views (TTE) at peak systole (Fig. 3b).
2. The points A and B represent the attachment locations of the leaflets (NCC and RCC) to the root wall (Fig. 3c). The imaginary line between points A and B serves as a reference line.
3. The resulting angle between the leaflets and the line AB (discussed in step 2) is measured at the peak systole time point of the parasternal long-axis plane view of the Doppler echocardiogram (TTE). The measured angles are the angular positions of each leaflet (NCC and RCC) at the fully open configuration (Fig. 3c).
4. Parasternal long-axis plane view intersecting with RCC and NCC is replicated in the computational domain. The point depicted in blue (Fig. 3d) represent the locations of intersection of the root attachment leaflet edge and the long-axis plane. The intersection of the parasternal long-axis plane view with the root is shown with A′ and B′ standing for the points A and B introduced in step 2. In the replicated plane drawn in the computational domain (Fig. 3d), the length of line A′B′ is matched to the length of the AB line in the TTE images.
5. Subsequently the C10 parameters of RCC and NCC leaflets are calibrated by matching the angular position at the peak systole time point of finite element results with angular position of the leaflets at the peak systole time point measured in step 3. Based on the initial C10 parameter, estimated boundary conditions calculated by the LPM and the prepared 3D geometry, a finite element simulation is performed to capture the peak systole time point (fully open configuration) of the RCC (or NCC) leaflet. The angular positions of the leaflets (RCC or NCC) are calculated at the peak systole time frame using A′B′ as the reference line, for each iteration of the assumed C10 parameter (Fig. 3e,f). The error function employed for the material calibration is defined as the difference between the angular position measured by TTE (Step 3) and the angular position calculated by finite element method. An iterative procedure is performed for both leaflets (NCC and RCC) with an error of less than one degree.
6. After the calibration of NCC and RCC leaflets, the geometric orifice area (GOA) is measured using parasternal short-axis view, only at the peak systole time point. The measured GOA is the geometrical parameter employed for the LCC material calibration using the parasternal short-axis rather than the previously used parasternal long-axis view (Fig. 3g).
7. The parasternal short-axis plane view passing through all three leaflets at the junction point of three leaflets is replicated in the computational domain. The sole factor affecting the GOA is the deformation of the LCC leaflet as the material calibration for RCC and NCC was conducted in prior steps (Fig. 3g).
8. As the material properties for RCC and NCC have been previously calculated, the remaining free parameter is the C10 parameters of the LCC leaflet. The C10 parameter of LCC leaflet is calibrated by matching the GOA at the peak systole time point of parasternal short-axis view in the finite element results with the GOA measured at peak systole time point of parasternal short-axis of Doppler echocardiography (TTE). In each iteration, peak-systole time frame of the finite element results is captured (Fig. 3h,i). To obtain C10 for the LCC leaflet, an error function is defined as the difference between GOA calculated in the computational domain and the measured GOA at the peak systole time from the parasternal short-axis view of TTE images. The relative error of this process is one percent.
9. Once all required parameters have been calibrated, the finite element simulation is performed on the full cardiac cycle. To reduce computational cost, finite element simulation intended for the calibration of each leaflet’s material properties (i.e., Steps 5 and 7) was performed solely to capture the fully open configuration of the aortic valve (i.e., peak systole).

For calibration, a bisection method was developed using PyCal software, ParaView, and SciPy libraries^134,138,139^. Specifically, the geometrical parameters of the finite elements are calculated via Python script within ParaView. Multiple iterations update the material's properties (i.e., C10) from an initial value of 0.3^140^, by invoking the PyCal library to update the CalculiX input file. Using the SciPy library, the bisection method-based mathematical calculations were performed.

### Patient-specific Doppler-based lumped-parameter modeling

We developed a non-invasive, Doppler-based, lumped parameter model ^38^ which includes several sub-models allowing for the analysis of complex and mixed valvular, ventricular, and vascular diseases including: (1) left atrium, (2) left ventricle, (3) aortic valve, (4) mitral valve, (5) systemic circulation, and (6) pulmonary circulation (Fig. 1). The calculations of the lumped-parameter model were validated against cardiac catheterization data (the instantaneous pressures in the aorta and LV) in patients with complex valvular, ventricular and vascular diseases with substantial inter- and intra-patient variability with a wide range of disease^38^. Moreover, some of the sub-models of the patient-specific lumped parameter algorithm have been used and validated previously^34,38,40–43,141–158^, with validation against in vivo cardiac catheterization in patients with valvular, ventricular and vascular diseases, in vivo MRI data in patients with AS, and in vivo MRI data in patients with mixed valvular diseases and coarctation.

### Statistics

Statistical analysis was performed using *Jamovi v.1.8.0*. Continuous variables were expressed as mean ± SD or median (interquartile range) as appropriate. Categorical data were presented as number (percentage). Pearson r or Spearman ρ were utilized to assess correlation between the continuous variables of the model. Comparisons between pre- and post-paired continuous variables were performed using Paired Student’s or Wilcoxon signed rank tests depending on normality. Statistical significance was considered when the p-value was less than 0.05.

## Results

### Validation: non-invasive patient-specific diagnostic framework (Doppler-based lumped-parameter model and Doppler-based 3-D non-linear finite element solver) vs. Clinical Doppler echocardiography data

#### Angular rotation

Figure 4 compares the angular rotation of the right coronary cusp (RCC) with the non-coronary cusp (NCC) using transesophageal echocardiographic data pre- and post-TAVR with the results from our Doppler-based diagnostic framework (Doppler-based lumped-parameter model coupled with finite element solver; Fig. 1) in 4 sample patients (out of 12 AS patients) at three varying time points throughout the cardiac cycle. The measured angular rotation using transesophageal echocardiography correlated well with the simulated results computed by our Doppler-based diagnostic framework in all patients (N = 12) investigated in this study with angular errors ranging from 0° (minimum) to 2° (maximum) in both pre- and post-TAVR.

#### Visible area

The visible area of all three aortic leaflets (left coronary cusp (LCC), RCC, NCC) is investigated in Fig. 4 based on the results from our Doppler-based diagnostic framework along with the measurements acquired by transesophageal echocardiography in 4 sample patients at three time points in the cardiac cycle, both pre- and post-TAVR. Based on the results, very strong agreements are shown between the Doppler-based diagnostic framework and transesophageal echocardiography in all patients (N = 12) investigated in this study with errors ranging from 0 to 5.7%, however, the majority are under 3.0% error.

### Current clinical assessment

#### Maximum Doppler pressure gradient (MIG)

Clinical assessment of AS for management and intervention decisions is performed based on the symptoms and hemodynamics metrics that focus locally and only on the aortic valve^34,38,159^. Based on the transvalvular pressure gradient, diagnosis and clinical decisions can be made^159^. According to the documented clinical Doppler echocardiography data, TAVR significantly decreased the maximum pressure gradient across the aortic valve (Fig. 11a, 52.2 ± 20.4 vs. 17.3 ± 13.8 [mmHg], p < 0.001). Individual data points reveal that, with the exception of Patient #11 whose pressure gradient during systole post-TAVR increased by 59%, all other patient maximum pressure gradients (11 out of the 12 patients (91%)) returned to a normal range (i.e., < 25 mmHg; reductions ranged from 25 to 86%).

#### Ejection fraction

Ejection fraction (EF), indicative of left ventricle contractility, measures the ability of the LV to pump blood with each heartbeat and is defined as $EF = \frac{EDV-ESV}{EDV}$; where EDV and ESV are end-diastolic volume and end-systolic volume, respectively^42^. Normal EF values reside above 41% for individuals with proper cardiac function^160^. Reduced left ventricular ejection fraction and low aortic valve pressure gradient during systole have been linked to poor long-term outcomes in patients with AS who undergo transcatheter aortic valve replacement^161^. We did not observe a significant increase in EF post-TAVR (Fig. 11b, 0.39 ± 0.11 vs. 0.41 ± 0.1, p = 0.6), three of the patients (Patients #11, #10 & #5) showed worsening EF. Patient #11’s ejection fraction worsened by 29% post-TAVR (41%), compared to pre-TAVR (53%) while Patient #5’s and Patient #10’s EFs post-TAVR were within the normal range (i.e., 41%) at 44% and 45%, respectively. Both Patient #5 and #10 experienced a reduction of 25% and 16%, respectively, compared to pre-TAVR values. TAVR did not raise EF values to normal levels for 5 out of 12 patients.

#### Ejection time and acceleration time

Few studies have considered ejection dynamic parameters such as ejection time (ET), acceleration time (AT) and ET/AT^162–165^. The ejection time (ET) is the time between the opening and closing of the aortic valve, while the acceleration time (AT) is the time it takes for an aortic valve to open and reach peak aortic jet velocity^164^. The parameters explaining ejection dynamics may be considered diagnostic parameters when there are inconsistencies between the aortic valve area and pressure gradient over the aortic valve during systole, which are used as common standards for evaluating aortic valve severity^165^. For prosthetic valves, acceleration time of greater than 100 ms is abnormal and AT/ET greater than 0.4 is indicative of an obstruction^166^. For native aortic valves, AT > 0.094 s and AT/ET > 0.35 might indicate severe aortic stenosis^165^. Figures 7, 10, S3 (Supplementary Material), and S6 (Supplementary Material), panel C, illustrate the change in ejection time and total cardiac duration between pre- and post-TAVR for patients #1, #3, #4, and #10, respectively. Patients #1, #4, and #10 exhibited improved ET and total cardiac duration, however, patient #12 exhibited a negligible improvement in ET and worsened cardiac duration post-TAVR. Following TAVR, there was a significant 16% reduction in ejection time (~ 91% of patients, Fig. 11c, 0.34 ± 0.04 vs. 0.29 ± 0.04 [s], p < 0.05). Despite this reduction in ejection time, two of the patients (Patients #11 and #3) showed a negligible change in ejection time post-TAVR. In one patient (Patient #12), ejection time worsened (increased by 22%) post-TAVR. Similarly, following TAVR we observed a significant 18% reduction in acceleration time (~ 91% of patients, Fig. 11d, 0.14 ± 0.02 vs 0.12 ± 0.02 [s], p < 0.05). However, Patient #11 showed an increase in acceleration time post-TAVR, relative to pre-TAVR. AT/ET did not significantly change following TAVR (Fig. 11e, 0.43 ± 0.06 vs. 0.42 ± 0.06, p = 0.6).

#### Diastolic dysfunction

The impaired relaxation of the left ventricle is often referred to as diastolic dysfunction^42,167^. In this study, the diastolic dysfunction was classified from Grade I to III based on the E wave to A wave ratio (E/A) from mitral inflow. In our group, diastolic dysfunction ranged from grade 1–3 in both pre- and post-intervention cases. The average grade for the 12-patient group increased from 2.25 pre-intervention to 2.42 post-TAVR. The condition for 3 (Patients #11, #7, #8) of the 12 patients worsened whereas only 1 patient (Patient #5) exhibited an improvement in diastolic dysfunction.

#### Paravalvular leakage

Paravalvular leakage is common post-operative complication following TAVR, due to imperfect sealing between the stent and the native aortic root. Almost all patients were diagnosed with some degree of PVL following procedure ([6/12] mild, [4/12] moderate and 1 severe).

### Global hemodynamics computed by Doppler-based diagnostic lumped-parameter model

In patients with aortic stenosis, the healthy instantaneous LV pressure and/or volume are altered which ultimately overloads the heart. We investigated metrics of cardiac function computed by our Doppler-based lumped-parameter model to determine the effects of TAVR on patient condition (Fig. 1, Panel a). The impacts of the TAVR on the aortic valve pressure gradient were not always accompanied by reduction in LV function parameter, e.g., LV workload, normalized LV workload to stroke volume and maximum LV pressure.

#### LV workload

LV workload represents the amount of energy delivered to the blood by the left ventricle in each cardiac cycle, plus the energy required to overcome the left ventricle's viscoelastic qualities, and is an effective metric for determining cardiac function^42^. The ideal LV workload is less than 1 [J] in healthy individuals with proper cardiac function^42,152,168^. LV workload was calculated as the area encompassed by the LV volume and LV pressure curves (Figs. 6, 9, S2 (Supplementary Material), S5 (Supplementary Material), panel C). TAVR is intended to reduce the LV workload by removing the severe aortic stenosis^1,34,38^. As shown in Figures S2 and S5, panel C, the LV workload drastically improved from 2.08 to 1.28 [J] and 2.57 to 1.26 [J] for patients #4 and #10, respectively. In contrast, Figs. 6 and 9, panel C show the worsened conditions with respect to LV workload for patients #1 and #3 whose workload increased from 1.23 to 1.43 [J] and 0.67 to 0.99 [J], respectively. The simulation results demonstrated that, despite a group level 13% reduction in LV workload (Fig. 12a, 1.8 ± 0.8 vs. 1.4 ± 0.4 [J], p = 0.05), only 8 of the 12 patients (~ 58%) had a reduction in LV workload post-TAVR. In four patients (Patients #1, #11, #3, #8) LV workload was not significantly reduced (improved) post-TAVR (< 5% reduction). In one patient (Patient #11), LV workload increased (worsened) by 54% post-TAVR.

#### Normalized LV workload

Normalized LV workload to stroke volume is the energy required to eject 1 ml of blood through the valvular-arterial system^42,144^. A significant 13% reduction was observed in normalized LV workload post-TAVR; as 9 out of the 12 patients (75%) showed an improvement in normalized LV workload post-TAVR (Figure S2(b), 0.017 ± 0.003 vs. 0.015 ± 0.002 [J/mL], p < 0.05). However, 3 patients (#1, #9 & #11) exhibited an increased normalized workload post-procedurally.

#### LV pressure

LV pressure is an important metric to measure and monitor when analyzing cardiac function as LV pressure overload can result in various cardiac diseases such as LV hypertrophy and failure. Maximum LV pressure observed in healthy individuals with proper cardiac function is below 120 [mmHg]^169^. Figures 6, 9, S2 and S5, panel C, show the LV pressure over the course of the cardiac cycle and have peaks of 136 [mmHg], 197 [mmHg], 179.8 [mmHg], and 190 [mmHg] pre-TAVR for patients #1, #3, #4, and #10, respectively. Post-TAVR, the maximum LV pressure observed in these patients were 144.96 [mmHg], 135.5 [mmHg], 121.7 [mmHg], and 127.76 [mmHg], respectively. It is evident through these results that the maximum LV pressure for patients #3, #4, and #11 significantly improved, however, Patient #1 had a worsened condition post-TAVR. Although, 9 of the 12 patients had a significant 18% reduction in maximum LV pressure and there was an overall group-level decrease (Figure S2(c), 166.4 ± 32.2 vs 131.4 ± 16.9 [mmHg]; p < 0.05) post-TAVR, Patient #12 showed a negligible difference (< 1%), while Patient #1 and Patient #11 had an increase in LV pressure (an increase of 6.6% and 42%, respectively). Despite the group level improvements in maximum LV pressure, only 5 of the 12 patients (41%) had a decrease in maximum aortic pressure (Fig. 12d).

#### Systemic arterial compliance

Arterial stiffening reduces the compliance of the systemic arterial system and is commonly linked to the development and progression of vascular and ventricular diseases^170^. Systemic arterial compliance (SAC = stroke volume index/pulse pressure) is an effective metric indicative of arterial hemodynamics and is inversely related to aortic stenosis morbidity risk^171^, where higher risk is correlated with a lower SAC value (less than 0.64 ml/m^2^/mmHg). There were minimal group-level changes in SAC following TAVR (Fig. 12e, 0.80 ± 0.30 vs. 0.75 ± 0.17 [ml/m^2^/mmHg], p = 0.6). While 7 of the 12 patients had a SAC value above 0.64 ml/m^2^/mmHg pre-TAVR, the simulation predicted that only 5 (patients #2, #3, #4, #8, #11) of the 12 patients would have had an improved SAC post-TAVR and 3 patients would have a negligible change (patients #5, #6, #11). The simulated change in SAC between pre- and post-TAVR states across all patients ranged from − 59% (Patient #9) to + 108% (Patient #8).

### Valve dynamics computed by non-invasive diagnostic framework (Doppler-based lumped-parameter model and Doppler-based 3-D non-linear finite element solver)

Aortic valve tissues experience time-dependent stress and displacement distributions as a result of transient loads. It is well known that stress can be a trigger for calcification and inflammation of native aortic valve tissues, and they can also cause failure and degeneration of transcatheter leaflets intensified by the immune system response and cyclic loadings^172^. Even though transcatheter leaflets are shown to have better compatibility with the immune and circulatory system, there is a remaining issue with their longevity which is far less than classical bioprosthetic valves^173^. Biomechanical factors resulting from hemodynamic loads are a common dominator of a variety of vascular diseases. Various mechanical metrics including 3-D von Mises stress (the deviatoric form of principal stress), 3-D major principal stress (the maximum value of the three principal stresses), as well as displacement magnitude resulted from our Doppler-based computational framework (Doppler-based lumped-parameter model coupled with Doppler-based 3-D non-linear finite element solver; Fig. 1) are described as follows:

#### 3-D von Mises stress

Von Mises stress refers to the derivative form of principal stress^174,175^. Figures 5, 8, S1 and S4 illustrate the 3-D motion of the valve throughout the entire cardiac cycle while displaying von Mises stress across all leaflets in both pre- and post-TAVR. Figures 6, 9, S2 and S5, panel A, show the transient 3-D distribution of von Mises stress over the aortic valve leaflets throughout the entire cardiac cycle for patients #1, #3, #4, and #10, respectively, in both pre- and post-TAVR states. Patient #1 (Fig. 6b) displayed elevated von Mises stress over the aortic valve specifically at the start of diastole when the valve is first closed, in both pre- and post-TAVR states. Patients #3, #4, and #10 all exhibited similar von Mises stress contours as there were elevated levels at both peak systole (open valve) and peak diastole (closed valve) pre-TAVR, however, post-TAVR von Mises stress contours displayed elevated levels solely in diastole (closed valve). The mean von Mises stress for all sample patients, with the exception of Patient#3, improved following the TAVR procedure. Post-TAVR, there was a significant 1.5 to 3-fold increase for each leaflet in group level maximum von Mises stress (at diastole; Fig. 13a).

#### 3-D major principal stress

As previously mentioned, it is hypothesized that abnormal stresses are a driving force behind the development of calcification and progression of aortic stenosis^2,12,19,21,30^. Stress components have different values depending on the desired coordinate system. When the area elements are considered such that the shear stress is eliminated, each element is solely loaded by normal stresses, known as principal stresses, which includes major principal stress, median principal stress, and minor principal stress^28,97^. We focused on the major principal stress, which is the maximum of the three principal stress components. Figures 7, 10, S3 and S6, panel B, illustrate the specific regions of time-averaged (over the full cardiac cycle) major principal stress of the individual leaflets in pre- and post-TAVR for patients #1, #3, #4, and #10, respectively. Overall, the major principal stress increased significantly post-TAVR particularly for leaflets near the right and non-coronary cusps by (100%, p < 0.01) and (81%, p < 0.01) respectively (Fig. 13b).

#### Displacement magnitude

In contrast to stress, which has long-term consequences, aortic valve tissue displacement could be a useful tool for monitoring aortic valve movements. Displacement is a vector that depicts the various components of each point's movement on the computational domain (leaflets of aortic valves) and could be a powerful tool for monitoring aortic valve movements^176^. All four sample patients showed a significant improvement in leaflet mobility as all leaflets increased in maximum displacement. The mean maximum displacement across all leaflets increased from 4.37 [mm] to 11.0 [mm], 4.77 [mm] to 14.0 [mm], 4.77 [mm] to 13.0 [mm], and 5.37 [mm] to 13.0 [mm] in patients #1, #3, #4, and #10, respectively. The results from our Doppler-based framework showed the maximum displacement (in the full cardiac cycle) increased for all leaflets by up to 3 times for leaflets near the left and right coronaries and by almost 1.36 times for the leaflet near the non-coronary cusp following TAVR on a group level basis (Fig. 13c, [4.3 ± 2.1, 4 ± 1.7, 7 ± 3.6] vs [13 ± 0.1.02, 13 ± 1.03, 13 ± 1.03], p < 0.001).

#### Patient-specific Doppler-based material properties of the valve leaflets

The stiffness or mechanical material properties of aortic valve leaflets describe the relationship between the displacement vector and stress tensor. Aortic stenosis is a condition in which the leaflets stiffen, resulting in less opening during ejection time. The TAVR procedure involves replacing stiff native aortic valve leaflets with prosthetic leaflets that have improved elasticity. Our framework can provide the dynamic behavior of aortic valve leaflets in addition to the asymmetric material properties of leaflets. Leaflet stiffness is illustrated in Figs. 7, 10, S3 and S6, panel C, which shows the patient-specific material properties for patients #1, #3, #4, and #10, respectively. Parameter C10 was used to evaluate leaflet stiffness which was evaluated for all aortic valve leaflets and represents the isotropic portion of the energy density function (Eq. 5). Increased C10 values represent an increased resistance of the leaflets to open when the left ventricle pressure exceeds the aorta pressure during systole^177,178^. We used Doppler images to calibrate each leaflet separately in our framework resulting in a specific C10 parameter for each leaflet. Pre-TAVR leaflet stiffness ranged from 0.51 to 1.48 [MPa] with a mean of 1.31 [MPa] for Patient #1 (Fig. 7c) which significantly reduced to range between 0.26 and 0.27 [MPa]. Similar results were observed for Patient #3 (Fig. 10c), pre-TAVR simulated results computed a range of 0.39–1.2 [MPa] with a mean of 0.90 [MPa] which improved to a range of 0.26–0.27 [MPa] across all leaflets. Furthermore, Patient #4 (Figure S3(c)) experienced a leaflet stiffness reduction from 0.4 to 1.66 [MPa] with a mean of 1.11 [MPa] pre-TAVR, to a range of 0.26–0.27 [MPa] post-TAVR. Finally, stiffness values of 0.51–1.48 [MPa] with a mean of 0.83 [MPa] were computed pre-TAVR for Patient #10 (Figure S6), which significantly decreased to 0.25–0.26 [MPa] across all leaflets post-TAVR. Moreover, on a group level basis, average stiffness among leaflets were calculated to be 0.90 ± 0.37 [MPa] (LCC), 1.03 ± 0.26 [MPa] (RCC), and 0.88 ± 0.43 [MPa] (NCC), pre-TAVR, and reduced to 0.26 ± 0.005[MPa] (LCC), 0.26 ± 0.006 [MPa] (RCC), and 0.26 ± 0.008 [MPa] (NCC), post-TAVR. For all patients, as shown in Fig. 13d stiffness among all leaflets was significantly reduced following TAVR, with 66, 74 and 52% mean reduction for each leaflet, respectively. Subsequently, this relates to a 2.8 times increase in geometric orifice area (Figure S3, 70.8 ± 11.6 vs 266 ± 43.8 [mm^2^], p < 0.001), and reduced the pressure gradient across the aortic valve. The open area between leaflets is known as the geometric orifice area (GOA)^179^. While effective orifice area (EOA) is a flow parameter, GOA which represents the available area for blood flow, which is slightly larger than EOA and is expected to increase post-TAVR^180^.

#### Aortic valve calcification

Aortic valve calcification is the most common cause of aortic stenosis^18,181,182^. Calcification evaluation and quantification can be used as a tool for diagnosis and monitoring of aortic valve disease^51,183^. The success of valvular intervention, such as TAVR, is strongly influenced by the presence of asymmetric calcification patterns^49^. Doppler echocardiography is limited in its use for calcification analysis, however, contrast CT imaging is a more powerful tool when evaluating calcium deposits^30,47,50^. Our Doppler-based lumped-parameter model coupled with a finite element solver can accurately quantify the stiffness and material properties of aortic valve leaflets. Despite the limitations of Doppler-echocardiography when analyzing calcification, our framework has shown a strong relationship between the stiffness measured with calcification of the valve. For instance, the more calcification present on the valve, the stiffer it will be. Figures 7, 10, S3 and S6, panel A, show the calcification as obtained from CT images which show a strong correspondence with the stiffness of the leaflets in Figs. 7, 10, S3 and S6, panel C, respectively. As shown in these figures, the new prosthetic valve structure mimics the healthy non-calcified native valve and we therefore observe greatly decreased stiffness levels after device implantation.

## Discussion

As an emerging alternative treatment strategy to surgery in patients with AS, transcatheter aortic valve replacement possesses several benefits, as well as risks^1^. The optimal function of the aortic valve is heavily influenced by the interaction between the blood flow and the structural properties of the valve^12,84^. Indeed, abnormal valve dynamics and abnormal hemodynamics are associated with adverse outcomes and must be quantified accurately to allow for accurate risk analysis and to potentially improve patient outcomes^27^.

Although medical imaging has made remarkable advancements and possess several benefits, however, none of these tools can quantify valve dynamics and (local and global) hemodynamics^38,184^. (1) *Doppler echocardiography (DE):* DE provides functional, real-time information regarding cardiac geometry, instantaneous flow and pressure gradient^185^. DE cannot evaluate local hemodynamics precisely and can not measure global hemodynamics and valve dynamics^186^; (2)* Phase-contrast magnetic resonance imaging (MRI):* MRI can provide local hemodynamics. However, MRI cannot measure any global hemodynamic and valve dynamics parameters and its use is limited in patients with implanted medical devices as they remain a major risk during the examination^151,187^; (3)* Computed tomography (CT):* Cardiac CT can evaluate valve calcification leaflet-specifically^188^. CT cannot measure any (local and global) hemodynamic parameters and can not measure valve dynamics. Such information has a high clinical importance for planning advanced treatments for patients with AS and TAVR.

We developed a Doppler-based computational-mechanics framework (Doppler-based patient-specific lumped-parameter model, 3-D non-linear finite element solver and 3-D Doppler-based geometry reconstruction) that can function as a diagnostic and monitoring tool for patients with AS in both pre- and post-intervention states at no risk to the patient. Our study brings the following insights:

### Doppler echocardiography pressure gradient is a poor indicator of aortic valve severity

Clinical assessment of AS for management and intervention decisions is performed based on the symptoms and hemodynamics metrics that focus locally and only on the aortic valve^34,38,159^. Based on the transvalvular pressure gradient, diagnosis and clinical decisions can be made^159^. According to the documented clinical Doppler echocardiography data, TAVR universally and significantly decreased the pressure gradient across the aortic valve. However, it is critically notable that reductions in transvalvular pressure gradient were not always accompanied by improvements in: (1) clinical metrics such as ejection fraction, ejection time, acceleration time and diastolic dysfunction classification; (2) LV hemodynamics metrics such LV workload, normalized LV workload and maximum LV pressure; (3) valve dynamics such as stress, distensibility and stiffness.

### TAVR does not always improve cardiac function metrics

Some patients, who underwent TAVR, experienced a significant improvement in terms of pronounced reverse LV remodeling and less congestive heart failure symptoms. However, the situation in some other patients worsened. In patients with aortic stenosis, the healthy instantaneous LV pressure and/or volume are altered which ultimately overloads the heart. We investigated metrics of cardiac function computed by our Doppler-based lumped-parameter model to determine the effects of TAVR on patient condition. The impacts of the TAVR on the aortic valve pressure gradient were not always be associated with reduction in LV function parameters, e.g., LV workload, normalized LV workload to stroke volume and maximum LV pressure. LV hemodynamics metrics worsened in some patients, and they were not significantly improved in the others. Furthermore, the presence of PVL particularly at the moderate and severe category may explain the reduction in EF in some cases and the increase in LV loads following procedure. Indeed, the global hemodynamic metrics could have a prognostic value for predicting and optimizing procedural outcomes and clinical decision support for managing patients post procedurally.

### TAVR does not always improve valve dynamics metrics

Aortic valve tissues experience time-dependent stress and displacement distributions as a result of transient loads. It is well known that stress can be a trigger for calcification and inflammation of native aortic valve tissues, and they can also cause failure and degeneration of transcatheter leaflets intensified by the immune system response and cyclic loadings^172^. Even though transcatheter leaflets are shown to have better compatibility with the immune and circulatory system, there is a remaining issue with their longevity which is far less than classical bioprosthetic valves^173^. Biomechanical factors resulting from hemodynamic loads are a common dominator of a variety of vascular diseases. Various mechanical metrics including 3-D von Mises stress (the deviatoric form of principal stress), 3-D major principal stress (the maximum value of the three principal stresses), as well as displacement magnitude resulted from our Doppler-based computational framework. Interestingly our early investigation implies that despite a marked improvement in the systolic function of the valve through improved leaflet mobility and increase in geometric orifice area, diastolic stresses post-TAVR elevated drastically. Indeed, biomechanical forces are the driving force behind the degeneration and failure of prosthetic heart valves and can be measured through this non-invasive framework. Which could indicate deterioration of the replacement valve^26^.

### Aortic valve calcification

Persistent monitoring and early diagnosis of aortic valve stenosis is key in the prevention and proper treatment planning for patients^189^. As the most common cause of AS, calcification in cardiovascular pathologies is commonly associated with adverse outcomes^18,181,182^. Aortic valve calcification scoring can be used as a diagnostic method to confirm disease severity as well as for intervention planning and prediction^51,183^. As a result, understanding the material properties and asymmetric physical features of native aortic valve leaflets can heavily influence and benefit clinical decision-making and surgical planning. Doppler echocardiography is limited in its applicability for analyzing calcification and cannot quantify valve calcification. However, CT calcification scoring is only indicated for patients with discordant echocardiographic diagnosis^50,159^ and even then the bulk CT score cannot directly describe valve biomechanics^50^. Therefore, using the developed non-invasive Doppler-based computational framework, we can quantify the stiffness and material properties for patients for whom CT is not indicated. This may have important clinical impacts regarding severe and non-severe calcific aortic stenosis grading and therefore a reclassification criterion for optimal intesrvention time^190,191^.

## Supplementary Information

Supplementary Information.
