# Author Correction: A Doppler-exclusive non-invasive computational diagnostic framework for personalized transcatheter aortic valve replacement

**DOI:** 10.1038/s41598-023-36288-w

**Published:** 2023-06-06

**Authors:** Nikrouz Bahadormanesh, Benjamin Tomka, Mohamed Abdelkhalek, Seyedvahid Khodaei, Nima Maftoon, Zahra Keshavarz-Motamed

**Affiliations:** 1 Department of Mechanical Engineering, McMaster University, JHE-310, Hamilton, ON L8S 4L7 Canada; 2 School of Biomedical Engineering, McMaster University, Hamilton, ON Canada; 3 Department of Systems Design Engineering, University of Waterloo, Waterloo, ON Canada; 4 Centre for Bioengineering and Biotechnology, University of Waterloo, Waterloo, ON Canada; 5 School of Computational Science and Engineering, McMaster University, Hamilton, ON Canada

Correction to: *Scientific Reports* 10.1038/s41598-023-33511-6, published online 17 May 2023

The original version of this Article contained an error in Reference 191,

191. Abdelkhalek, M. *et al.* Patterns and structure of calcification in aortic stenosis. *JACC Cardiovasc. Imaging.* https://doi.org/10.1016/j.jcmg.2023.02.011 (2023).

now reads:

191. Abdelkhalek, M. et al. Patterns and structure of calcification in aortic stenosis: An approach on contrast-enhanced CT images. *JACC Cardiovasc. Imaging*. https://doi.org/10.1016/j.jcmg.2023.02.011 (2023).

The original Article has been corrected.

