# Supplementary material for: A Doppler-exclusive non-invasive computational diagnostic framework for personalized transcatheter aortic valve replacement
Source: Sci Rep. 2023 May 17;13:8033. doi: 10.1038/s41598-023-33511-6 (PMC10192526; doi:10.1038/s41598-023-33511-6)
Supplement: Supplementary file 1 — Supplementary Information. [file 41598_2023_33511_MOESM1_ESM.docx]

Supplementary Material

**Figure S1**


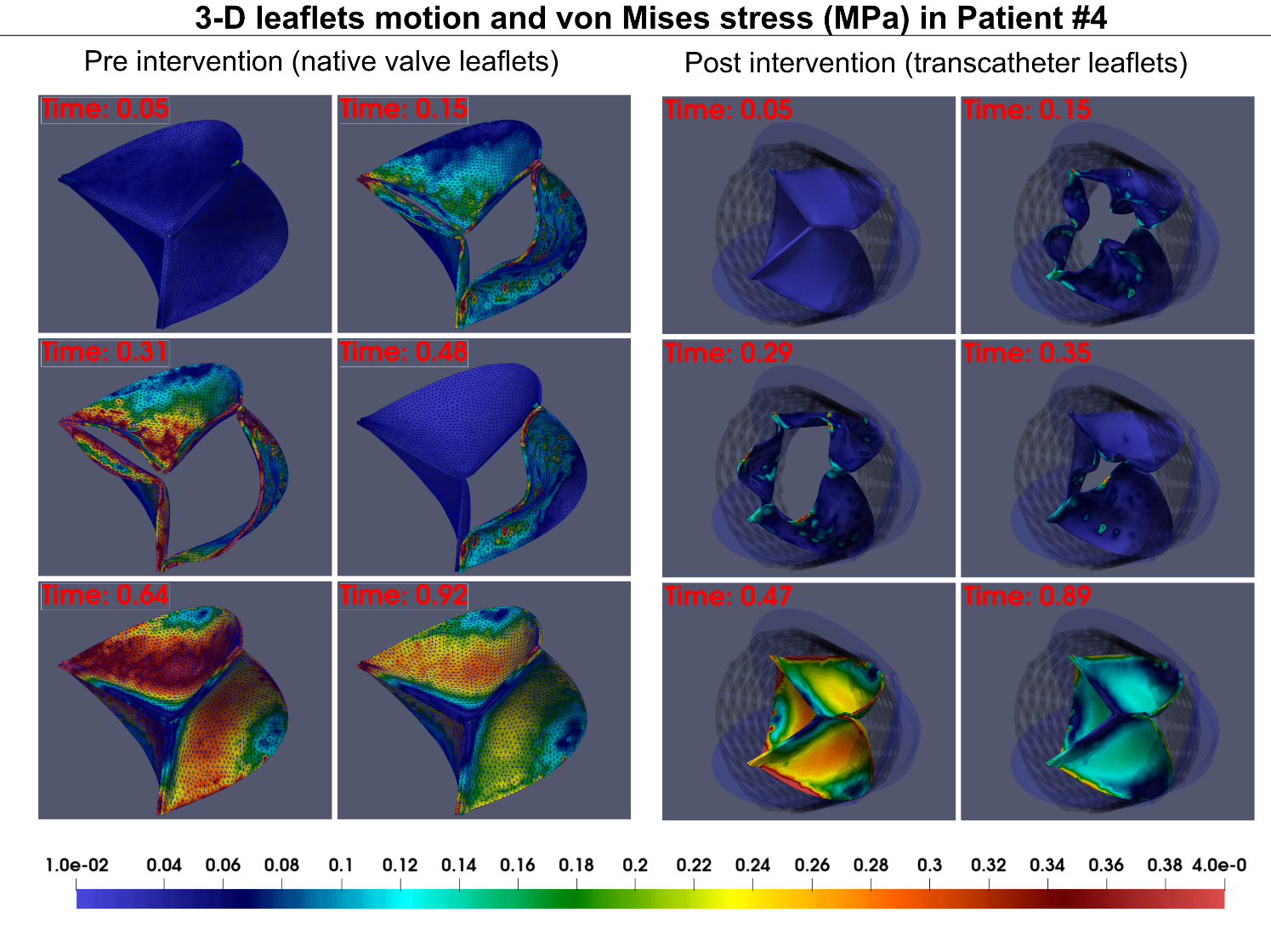


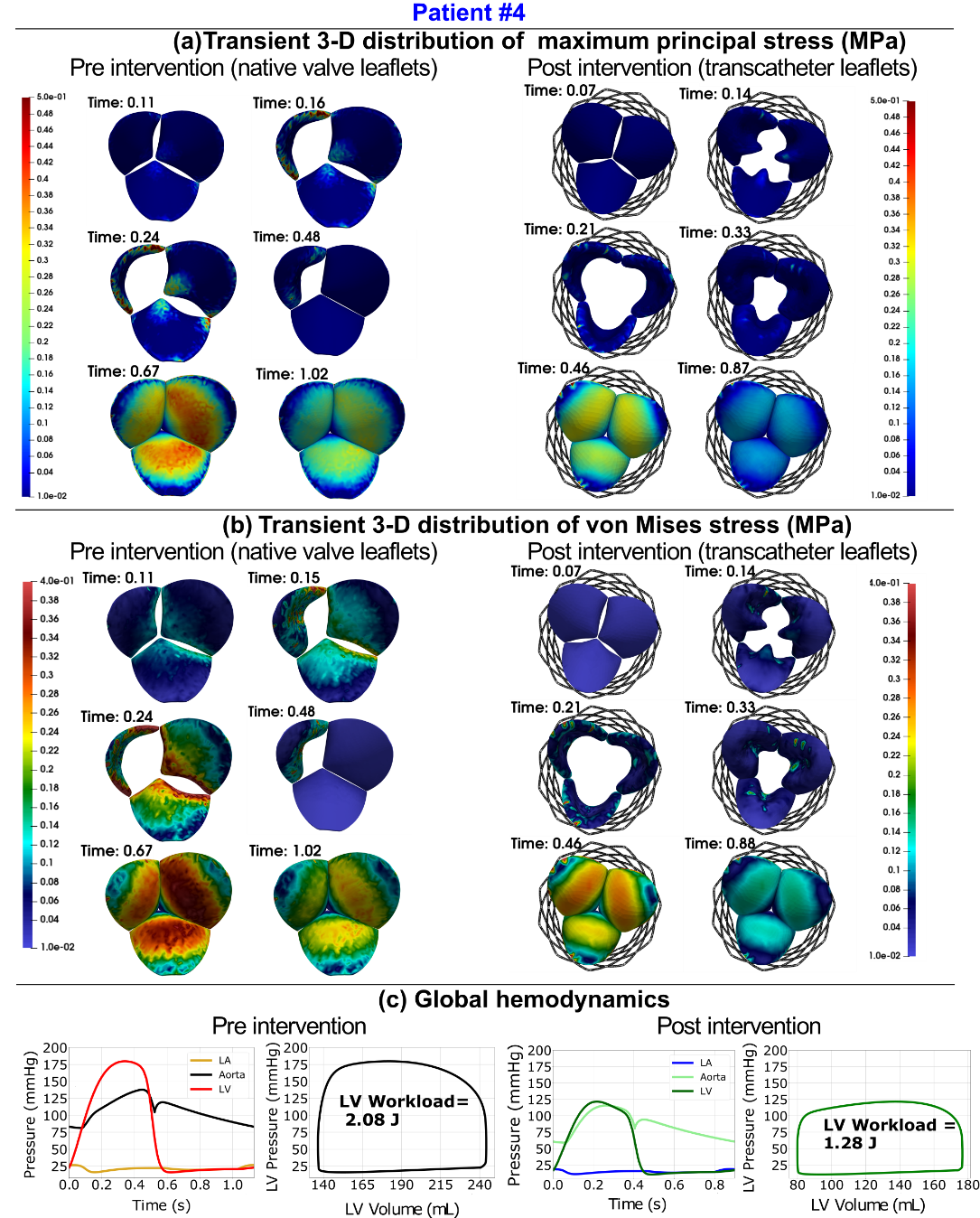
**Figure S2**

**Figure S3**


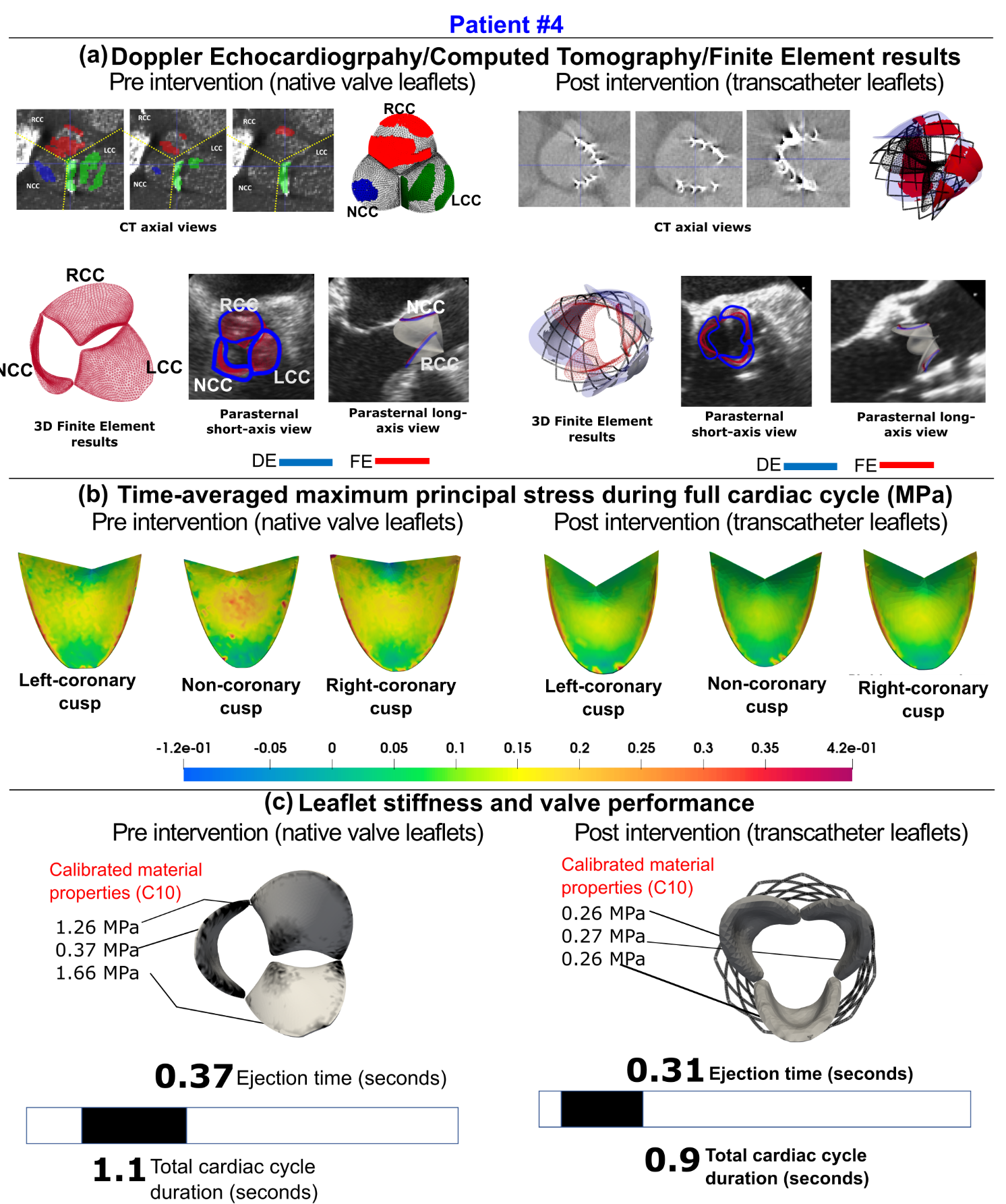


**Figure S4**


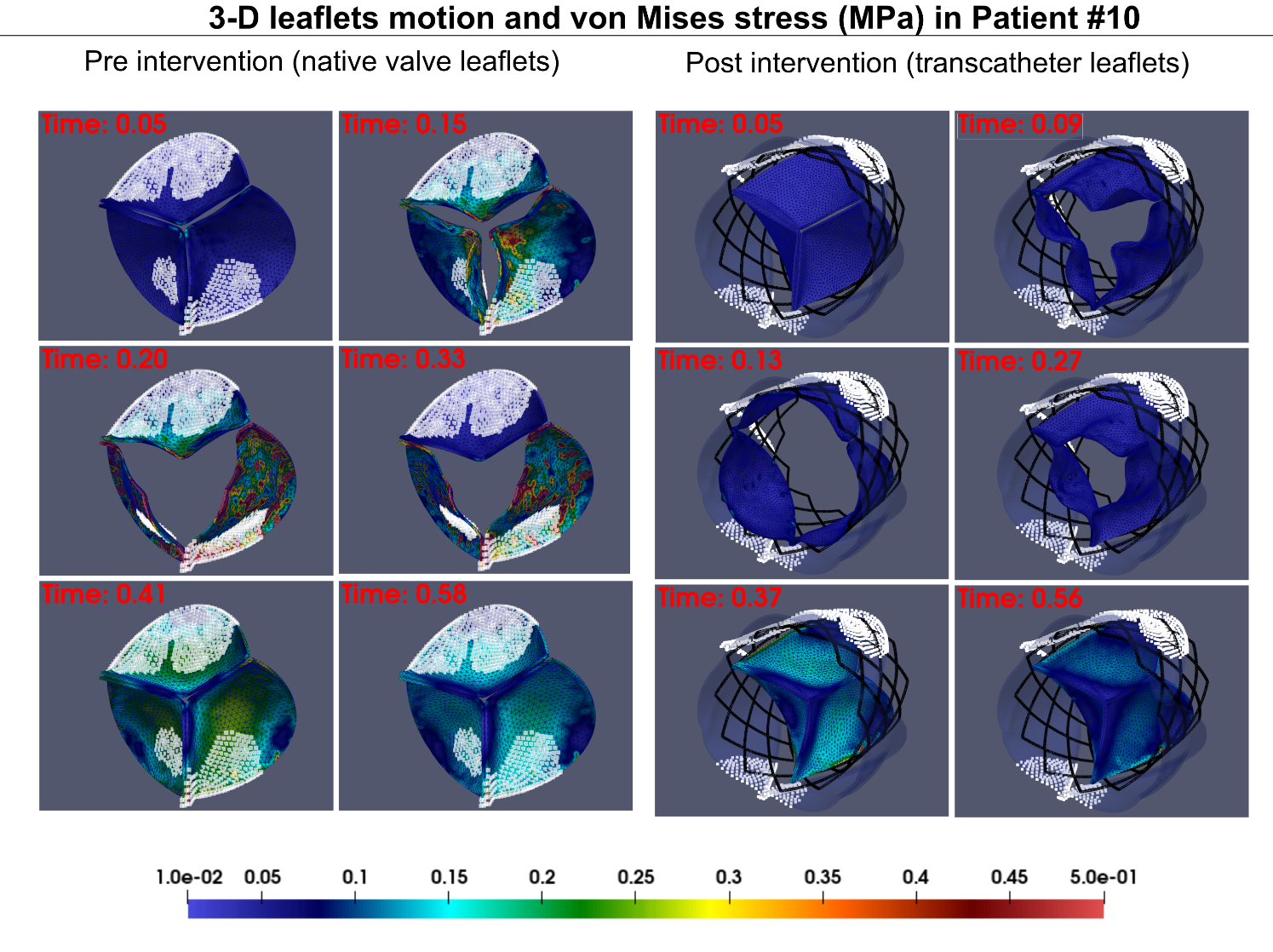


**Figure S5**
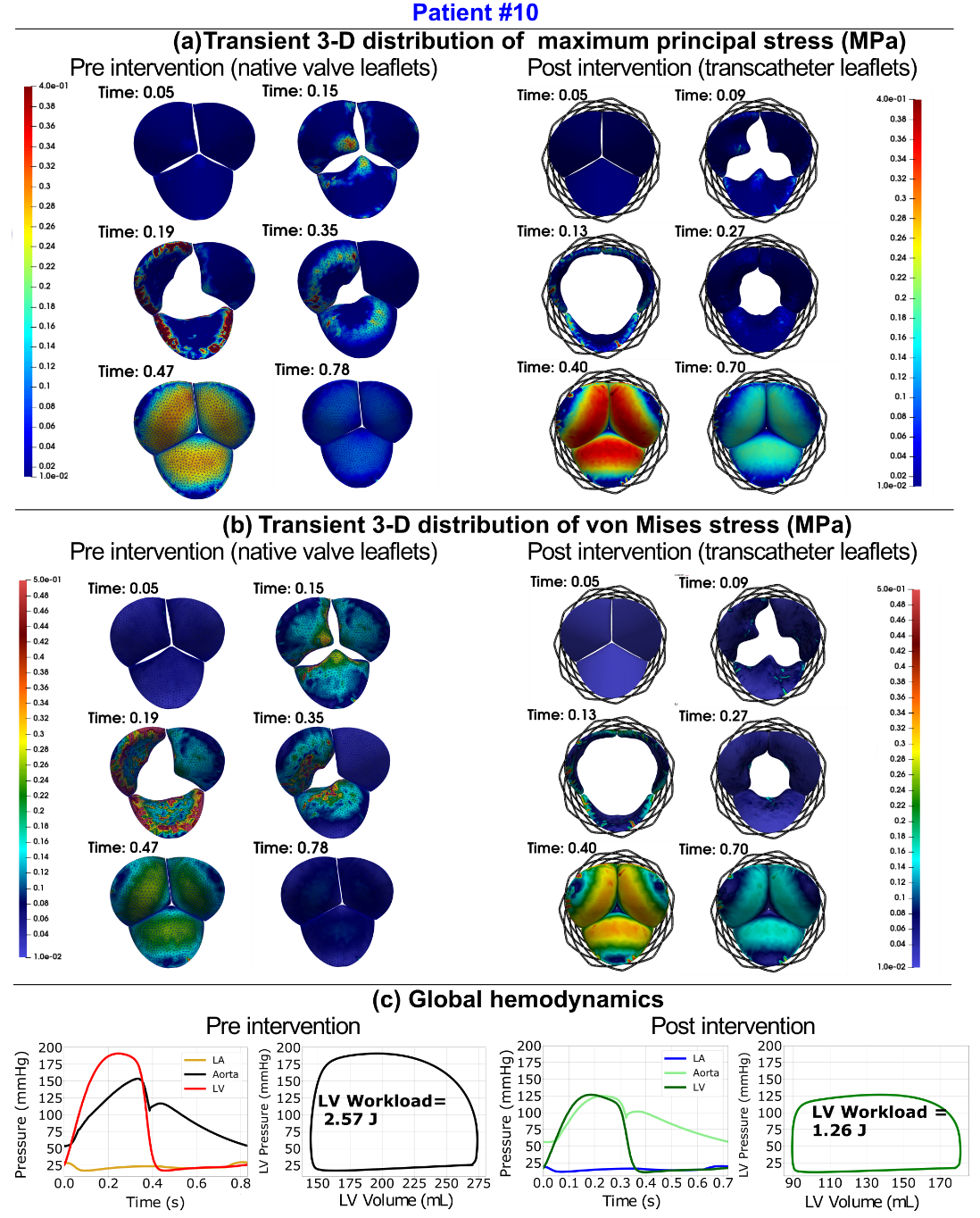


**Figure S6**


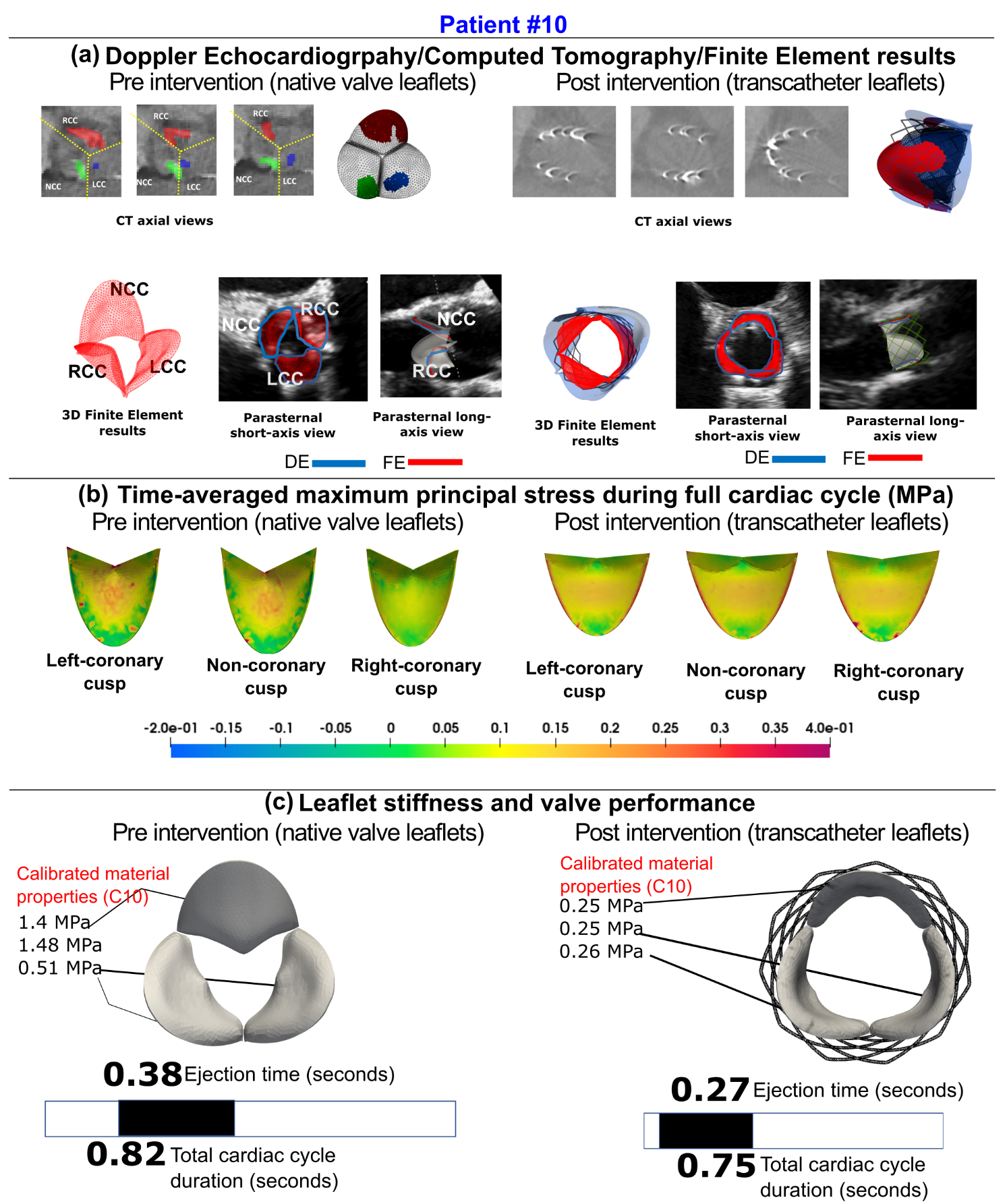


**Figure S7**


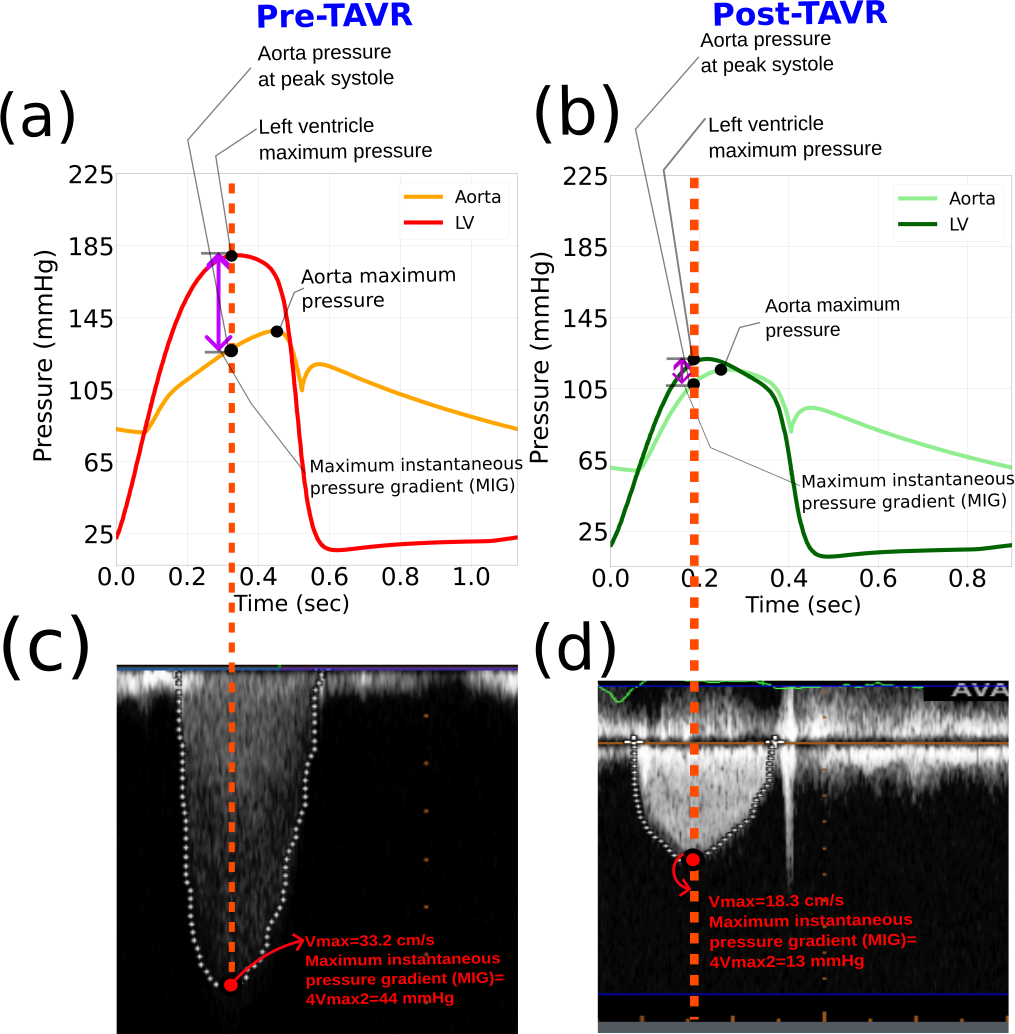


**Figure S8**


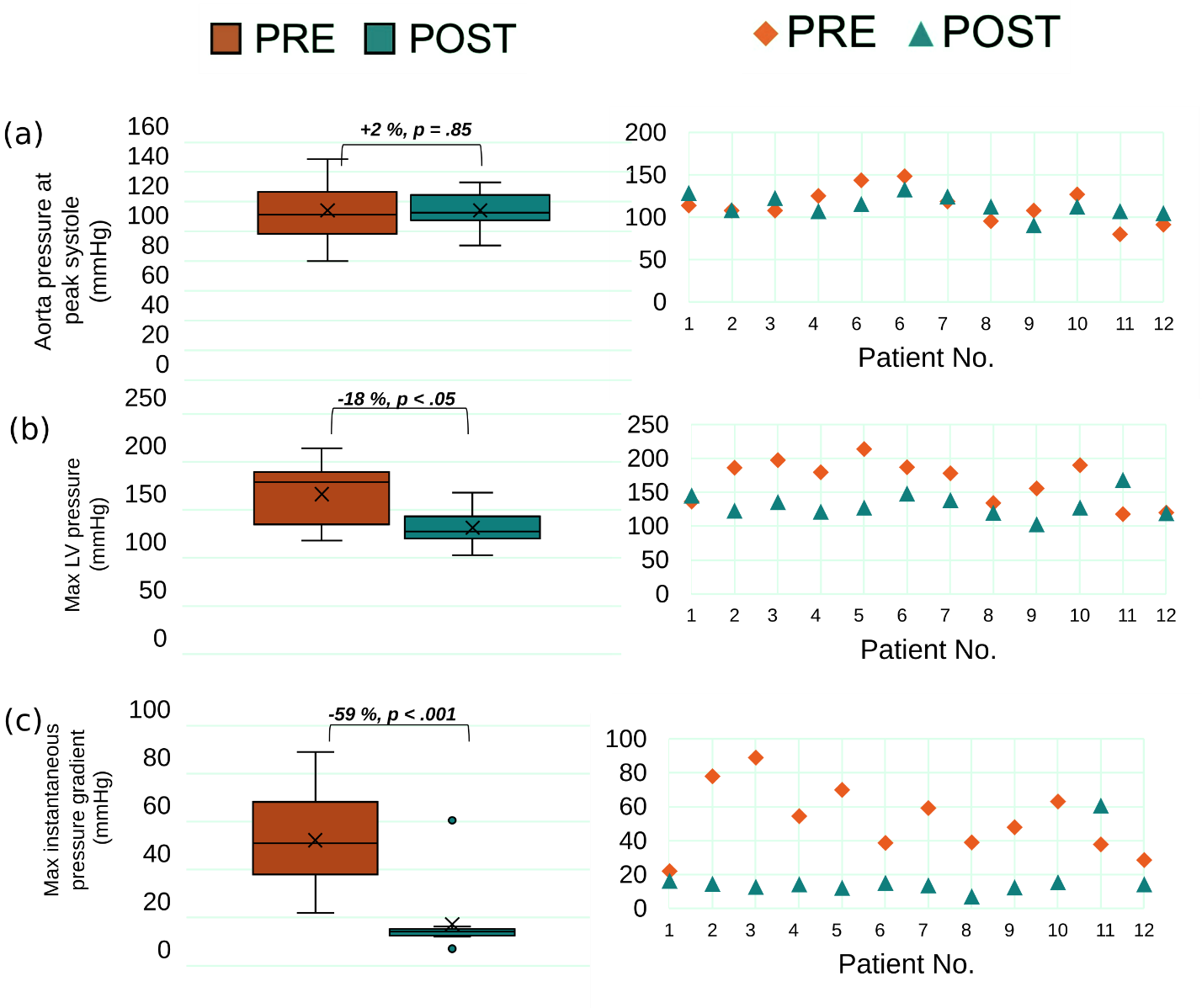


**List of Figures**

**Figure S1.** **3D motion and 3D distribution contours of von Mises stress in patient #4 at six time points throughout the cardiac cycle in both pre- and post-intervention states.** Using our framework, we estimated 3D deformation of aortic valve leaflet during full cardiac cycle as well as the von Mises stress distribution.

**Figure S2. Changes in valve dynamics and global hemodynamics in patient #4 between baseline and 90-day post-TAVR.** (a) Transient distribution of major principal stress at six time phases of the cardiac cycle: early systole (start of valve opening), early to peak systole (valve opening), peak systole (fully open), peak to late systole (valve closure), early diastole (closed configuration) and late diastole (closed configuration); (b) Transient distribution of the von Mises stress at six time phases of the cardiac cycle: early systole(start of valve opening), early to peak systole(valve opening), peak systole (fully open), peak to late systole (valve closure), early diastole (closed configuration) and late diastole (closed configuration); (c) *Global hemodynamics*: LV workload; aorta and LV pressures in both pre- and post-intervention states. ***Patient #4***: *Pre-TAVR*: severe aortic stenosis (EOA=0.7 cm^2^), coronary artery disease and hypertension, mild mitral regurgitation, mild aortic regurgitation, ejection fraction: 10%, brachial pressures: 80 and 140 mmHg; *Post-TAVR*: aortic valve (EOA=2.0 cm^2^), mild aortic regurgitation, ejection fraction: 8%, brachial pressures: 61 and 115 mmHg.

**Figure S3. Comparison of doppler-based finite element results with computed tomography and / leaflet-specific time-averages major principal stress/ Doppler-based calibrated material properties- Patient #4.** (a) Computed tomographic and doppler echocardiographic images compared to the results of the doppler-based finite element solver; (b) The time-averaged maximum principal stress on all native aortic valve leaflets pre-intervention and all transcatheter valve leaflets post-intervention; (c) Results of the doppler-based framework illustrating the material properties and leaflet stiffness as well as performance features such as ejection time and cardiac cycle duration.

**Figure S4.** **3D motion and 3D distribution contours of von Mises stress in patient #10 at six time points throughout the cardiac cycle in both pre- and post-intervention states.** Using our framework, we estimated 3D deformation of aortic valve leaflet during full cardiac cycle as well as the von Mises stress distribution. The regions covered with white points are representing the calcified areas visualized manually by using multi-slice CT images.

**Figure S5.** **Changes in valve dynamics and global hemodynamics in patient #10 between baseline and 90-day post-TAVR.** (a) Transient distribution of major principal stress at six time phases of the cardiac cycle: early systole (start of valve opening), early to peak systole (valve opening), peak systole (fully open), peak to late systole (valve closure), early diastole (closed configuration) and late diastole (closed configuration); (b) Transient distribution of the von Mises stress at six time phases of the cardiac cycle: early systole(start of valve opening), early to peak systole(valve opening), peak systole (fully open), peak to late systole (valve closure), early diastole (closed configuration) and late diastole (closed configuration); (c) Global hemodynamics: LV workload; aorta and LV pressures in both pre- and post-intervention states. Patient #10: Pre-TAVR: severe aortic stenosis (EOA=0.8 cm2), type 2 diabetes mellitus, moderate aortic regurgitation, coronary artery disease and hypertension, ejection fraction: 52%, brachial pressures: 59 and 164 mmHg; Post-TAVR: aortic valve (EOA=2.0 cm2), type 2 diabetes mellitus, ejection fraction: 45%, brachial pressures: 58 and 127 mmHg.

**Figure S6. Comparison of doppler-based finite element results with computed tomography and / leaflet-specific time-averages major principal stress/ Doppler-based calibrated material properties- Patient #10.** (a) Computed tomographic and doppler echocardiographic images compared to the results of the doppler-based finite element solver; (b) The time-averaged maximum principal stress on all native aortic valve leaflets pre-intervention and all transcatheter valve leaflets post-intervention; (c) Results of the doppler-based framework illustrating the material properties and leaflet stiffness as well as performance features such as ejection time and cardiac cycle duration.

**Figure S7.** Detailed analysis of pressure and pressure gradient changes in both pre- and post-TAVR status for a sample patient (Patient #4). (a) Simulated transient left ventricle and aorta pressures using our Doppler-based patient-specific lumped-parameter model in pre-TAVR status; (b) Simulated transient left ventricle and aorta pressures using our Doppler-based patient-specific umped-parameter model in post-TAVR status; (c) Measured velocity and calculated clinical Doppler pressure gradient using Doppler echocardiography data in pre-TAVR status (ΔP=4V^2^_MAX_; V_MAX_: maximum velocity); (d) Measured velocity and calculated Doppler pressure gradient using clinical Doppler echocardiography data in post-TAVR status.

**Figure S8.** Changes in clinical assessment and simulated pressures using our Doppler-based lumped-parameter model (pre TAVR *vs.* post-TAVR; n = 12). (a) Simulated aorta pressure at peak systole using our lumped parameter model; (b) Simulated left ventricle pressure at peak systole using our lumped parameter model; (c) Doppler pressure gradient using clinical Doppler echocardiography data.
